# Integrative stratification of response to immunotherapy using host and microbial signatures in patients with advanced non-small cell lung cancer

**DOI:** 10.1186/s13073-026-01736-5

**Published:** 2026-08-06

**Authors:** Jii Bum Lee, Junwon Yang, Mauricio Chalita, Yeonjae Jung, Haangik Park, Myungja Ro, Hyun Kim, Eunchong Yang, Soeun Choi, Minyoung Kim, Arim Min, Bo-Eun Kwon, Jieun Im, Hyunjeong Kim, Hyunkyung Park, Sun Min Lim, Min Hee Hong, Sein Park, Yeong Ouk Kim, Jongsik Chun, Hyun-Seok Oh, Byoung Chul Cho

**Affiliations:** 1 Division of Medical Oncology, Department of Internal Medicine, Yonsei Cancer Center, Yonsei University College of Medicine, 50 Yonsei-Ro, Seodaemun-Gu, Seoul, 03722 Republic of Korea; 2 CJ Bioscience, Inc., 7th Floor, Grand Central B, 14 Sejong-Daero, Jung-Gu, Seoul, 04527 Republic of Korea; 3 Interdisciplinary Program in Bioinformatics, Seoul National University, Seoul, Republic of Korea; 4 Severance Biomedical Science Institute, Yonsei University College of Medicine, Seoul, Republic of Korea

**Keywords:** Gut microbiome, Immune checkpoint inhibitors, Non-small cell lung cancer

## Abstract

**Background:**

The gut microbiome has been linked to responses to immune checkpoint inhibitors (ICIs), yet longitudinal multi-omics studies remain limited.

**Methods:**

Here, we serially collected gut microbiome data and performed multi-omics analyses, including RNA sequencing (RNA-seq) and whole-exome sequencing (WES), on samples from 92 patients with advanced non-small cell lung cancer (NSCLC) treated with pembrolizumab, to identify factors contributing to treatment response. We compared gut microbiome data with those of a healthy cohort (*n* = 92) and examined changes in gut microbial signatures during immunotherapy. Gut microbiome data from publicly available ICI-treated cohorts (*n* = 728) were also used together with our own cohort to develop and validate a score for classifying response to ICIs.

**Results:**

Responders (defined as complete response, partial response, or stable disease of at least 6 months) were enriched for *Faecalibacterium*, while non-responders were enriched for *Lactobacillus* and *Escherichia*, with these taxa showing specific associations with dietary fiber intake and antibiotic use. Dynamic shifts in dysbiotic signatures were observed throughout treatment and disease progression, with dissimilarity from the healthy cohort decreasing after treatment only in responders but increasing at progression in both responders and non-responders. We also developed a cross-population score based on the ratio of responder- to non-responder-associated microbial markers. This score showed a significant positive correlation with the index reflecting gut microbial health status and improved response stratification when combined with tumor mutational burden (TMB).

**Conclusions:**

These findings highlight the importance of characterizing dysbiosis using gut microbial signatures, which act along a distinct and complementary axis to explain responses to ICIs.

**Supplementary Information:**

The online version contains supplementary material available at https://doi.org/10.1186/s13073-026-01736-5.

## Background

Immune checkpoint inhibitors (ICIs) have revolutionized treatment paradigm in various solid tumors, yet only a subset of patients achieves durable benefit [1]. Growing evidence suggests that the gut microbiome is one of the key factors influencing the efficacy of ICIs. Associations between the gut microbiome and the efficacy of ICI were first reported in mouse models in 2015 [2, 3] and later identified in patients in 2018 [4–6]. These studies demonstrated that fecal microbiota transplantation (FMT) from ICI responders into germ-free mice improved tumor control [4–6]. More recent clinical trials have shown that FMT using stool samples from healthy donors [7] or ICI responders [8, 9] can enhance the efficacy of ICIs in patients with solid tumors. Nonetheless, diverse microbial markers of response have been reported across cohorts [4–6], prompting efforts to integrate data across studies to identify consistent microbial signatures [10, 11] or to define response-associated bacterial communities rather than individual taxa [12]. Previously, most studies have focused on Western populations, and few longitudinal studies have investigated gut microbiome dynamics during treatment with ICIs [13]. The diverse factors influencing response to ICI also highlight the importance of integrating multi-omics data with gut microbiome data.

In this study, we collected gut microbiome samples from 92 patients with advanced non-small cell lung cancer (NSCLC) in Korea, where the typical diet differs from that of Western countries, in order to identify microbial features associated with response to pembrolizumab. We also compared data from NSCLC cohort with those of a healthy cohort in Korea and investigated the dynamics of gut microbial signatures during treatment with immunotherapy. Next, we defined a bacterial ratio score and assessed its association with response to ICI across cohorts, including our cohort and publicly available cohorts from Western countries. We further integrated this score with tumor mutational burden (TMB) to identify response stratification to ICIs.

## Methods

### Study design and cohort description

Between September 2022 and November 2023, patients with histologically confirmed, treatment-naïve, stage IV NSCLC without *EGFR* or *ALK* mutations were enrolled. General inclusion criteria included ages 18 years or above, Eastern Cooperative Oncology Group (ECOG) performance status of 0 or 1, adequate organ function, and at least one measurable lesion according to the Response Evaluation Criteria in Solid Tumors version 1.1 (RECIST v1.1). Patients were excluded if they had prior exposure to PD-1 or PD-L1 inhibitors, previous allogeneic organ transplant, or history of major surgery within 28 days before treatment. This study was conducted in accordance with the International Conference on Harmonization Guidelines on Good Clinical Practice and the Declaration of Helsinki. All participants provided written informed consent, and the protocol was approved by the Institutional Review Board of Yonsei Cancer Center (IRB 4–2022-0355).

Tumor PD-L1 expression was assessed using the PD-L1 IHC 22C3 pharmDx assay (clone 22C3, Dako North America, Inc., Carpinteria, CA, USA) on formalin-fixed, paraffin-embedded (FFPE) samples obtained at diagnosis. Patients whose tumors had a PD-L1 tumor proportion score (TPS) ≥ 50% received pembrolizumab 200 mg for up to 35 cycles or pembrolizumab plus chemotherapy at the discretion of the investigator. Patients with TPS < 50% and non-squamous histology received pembrolizumab plus four cycles of carboplatin (area under the concentration–time curve 5) and pemetrexed (500 mg/m^2^). Patients with TPS < 50% and squamous histology received pembrolizumab plus four cycles of carboplatin (area under the concentration–time curve 5) and paclitaxel (200 mg/m^2^). All treatments were administered intravenously in 3-week cycles. Premedication included folic acid, vitamin B12, and glucocorticoids for patients receiving pemetrexed, and glucocorticoids plus antihistamines for patients receiving paclitaxel. Dose reductions were not permitted for pembrolizumab, but treatment could be interrupted, delayed, or discontinued based on tolerability. Dose reductions for pemetrexed, paclitaxel, and carboplatin were permitted at two levels of dosage, and dose interruption and discontinuation were followed as per the protocol. Patients continued treatment until disease progression, unacceptable adverse events, or a decision by either the investigator or the patient to discontinue.

Chest and abdominal computed tomography (CT) scans were performed at weeks 6 and 12, then every 9 weeks thereafter for response evaluation according to RECIST v1.1. Patients were followed every 12 weeks to assess survival. Treatment-related adverse events were reported according to the Common Terminology Criteria for Adverse Events (CTCAE) v5.0. Responders were defined as patients who achieved a complete response, partial response, or stable disease lasting at least 6 months. Non-responders were defined as those with progressive disease or stable disease lasting less than 6 months. PFS was measured from treatment initiation to progressive disease or death. OS was measured from treatment initiation to death from any cause. The data cutoff was January 2025, and patients without a known date of death were censored at their last follow-up.

### Assessment of dietary and nutrient intake

The habitual dietary and nutrient intake patterns of patients were assessed using a semi-quantitative FFQ, which was collected at screening (*n* = 49) and at progression (*n* = 20). The FFQ was based on the 7th Korea National Health and Nutrition Examination Survey (KNHANES), conducted by the Korea Disease Control and Prevention Agency (KDCA). This FFQ consists of 112 items and was developed and validated to investigate the habitual diet of Koreans [14, 15]. Nutrient intake was calculated based on the nutrient composition database established by the KDCA, which was constructed using the 8th edition of the Food Composition Table published by the Korean Rural Development Administration.

### Shotgun and amplicon sequencing of stool samples

Stool samples for both shotgun and amplicon sequencing were collected at screening (*n* = 92), after C2D1 (*n* = 82), and at progression (*n* = 36). Total DNA was extracted from stool samples following the manufacturers’ protocols using either the FastDNA Spin Kit (MP Biomedicals) or the Maxwell RSC PureFood GMO and Authentication Kit (Promega).

For shotgun sequencing, extracted DNA concentrations were quantified using a Qubit 2.0 fluorometer (Invitrogen). Sequencing libraries were prepared with the NEBNext Ultra II FS DNA Library Prep Kit for Illumina (NEB) according to the manufacturer’s protocol. Shotgun sequencing was conducted at CJ Bioscience, Inc., using either an Illumina NextSeq or NovaSeq system.

For amplicon sequencing, the V3–V4 regions of the 16S rRNA gene were amplified by polymerase chain reaction (PCR) using primers 341 F (CCTACGGGNGGCWGCAG) and 805R (GACTACHVGGGTATCTAATCC). PCR amplification conditions included an initial denaturation at 95 °C for 3 min, followed by 25 cycles of denaturation at 95 °C for 30 s, primer annealing at 55 °C for 30 s, and extension at 72 °C for 30 s, with a final elongation at 72 °C for 5 min. PCR products were verified using 1% agarose gel electrophoresis and visualized on a Gel Doc system (Bio-Rad). Amplicons were purified, and short non-target fragments were removed with the ProNex Size-Selective Purification System (Promega). The quality and size of the PCR products were assessed using a Bioanalyzer 2100 (Agilent) with a DNA 7500 chip. The pooled amplicons were sequenced using an Illumina MiSeq system at CJ Bioscience, Inc., following the manufacturer’s instructions.

### Taxonomic profiling of stool samples

Shotgun sequencing data were taxonomically profiled using the EzMx platform [16]. The potential presence of bacterial and archaeal species in each shotgun sequencing dataset was assessed using Kraken2 [17] and a pre-built core gene database [18], which contains k-mers (*k* = 35) derived from reference genomes obtained from the EzBioCloud database [16]. Subsequently, each shotgun sequencing dataset was mapped against a custom Bowtie2 database [19], constructed using core genes and genomes from species identified in the previous step. SAMtools [20] was used to convert and sort the resulting BAM files, and BEDTools [21] was employed to determine the coverage of mapped reads. The resulting profiles were used to create phyloseq objects [22] for further downstream analysis, and read counts were rarefied to the minimum library size (334,615 reads) for diversity analysis.

Alpha diversity within the cohort was determined using amplicon sequencing data processed with QIIME2 (v2022.11) [23], followed by rarefaction to 7,390 reads. Primer sequences were trimmed with the cutadapt plugin [24], and untrimmed reads were discarded. Paired-end reads were processed and denoised with the dada2 plugin [25]. The resulting amplicon sequence variants (ASVs) were classified against the EzBioCloud database [16] using the classify-consensus-vsearch pipeline with a 97% identity threshold and 80% coverage [26, 27]. Additionally, the gut microbiome index (GMI) was calculated from the amplicon sequencing data according to previously described methods [28].

### Bacterial categories grouped by functional roles

The bacterial categories used in this study were grouped by functional roles. The cumulative relative abundance of 25 bacterial genera was used to quantify the abundance of butyrate-producing bacteria. These genera include those used as butyrate-producing bacteria in a previous study [29], as well as additional genera identified through an in-house analysis using gutSMASH [30] with bacterial genome data from the EzBioCloud database [16]. Probiotic bacteria in this study correspond to the 19 probiotic species listed by the Korean Ministry of Food and Drug Safety (MFDS). Inflammation-inducing bacteria correspond to genera within the Enterobacteriaceae family, which are known to induce inflammation [31].

### Machine learning for response classification

Species-level profiles were used to assess associations with response using a random forest model implemented with scikit-learn [32]. When the same dataset served as both the training and test set, three-fold stratified cross-validation was repeated 100 times. Conversely, when different datasets were used for training and testing, the entire procedure was repeated 100 times.

### Gene cluster detection for assembled contigs

Shotgun sequencing data were assembled using the nf-core/mag pipeline (v2.2.1) [33]. The resulting contigs were analyzed for gene cluster detection using antiSMASH [34], gutSMASH [30], and dbCAN [35], which respectively target secondary metabolic gene clusters, primary metabolic gene clusters, and carbohydrate-active enzymes (CAZymes). For each tool, the relative abundance of detected gene clusters was computed by dividing the total depth of contigs containing a specific gene cluster by the total depth of contigs containing all gene clusters identified by that tool. Major contributing bacteria at the genus level were determined based on the relative abundance of contigs assigned to metagenome-assembled genomes (MAGs) obtained from the binning process, utilizing the taxonomy assigned to each MAG. Contigs not assigned to MAGs or assigned to MAGs without identified taxonomy were categorized as unclassified.

### Metagenomic data collection for the healthy Korean cohort

A total of 348 healthy Korean subjects with quality-controlled, paired shotgun and amplicon sequencing data from stool samples were obtained from the previously conducted CJ Bioscience’s healthy Korean cohort (IRB 2016–06–140) [28]. Of these, 92 subjects were selected through propensity score matching with the NSCLC cohort based on age, BMI, and sex for comparative analysis. For diversity analyses comparing the NSCLC and healthy Korean cohorts, read counts were rarefied to 154,432 reads, corresponding to the minimum library size of the healthy Korean cohort.

### Metagenomic data collection for publicly available cohorts treated with ICI

A total of 982 metagenomic samples were obtained from nine publicly available cohorts treated with ICI [5, 6, 10, 11, 36–39]. Of these, nine samples containing fewer than 150,000 reads after profiling were excluded. Subsequently, we computed the Jensen–Shannon distance (JSD) at the genus level between pairs of quality-filtered samples. Twenty-nine pairs of samples from the Derosa_2022_NSCLC and Routy_2018_NSCLC cohorts exhibited JSD values below 0.1; thus, 29 samples from Derosa_2022_NSCLC were removed. Additionally, one pair of samples from Spencer_2021_MEL (SRR16554778 and SRR16554786), despite originating from different subjects, also had a JSD below 0.1 and was therefore excluded. From the remaining 942 samples, we classified complete and partial responses as responders and progressive disease as non-responders, excluding 214 samples labeled as stable disease or indistinguishable in the discovery cohorts (*n* = 511). For publicly available metadata that did not differentiate stable disease, we retained their original response classification and included them in the validation cohorts (*n* = 217). The Peters_2019_MEL cohort did not explicitly categorize patients as responders or non-responders; therefore, patients with more than six months of PFS were classified as responders, while the others were classified as non-responders.

### Calculation of bacterial ratio score

To identify cross-cohort gut microbial signatures, a random-effects meta-analysis was conducted using the discovery cohort. A total of 19 responder-enriched markers and 23 non-responder-enriched markers were identified at the species level (*P* value < 0.05; found across all six cohorts). The bacterial ratio score was calculated as the sum of responder-enriched marker abundances divided by the sum of non-responder-enriched marker abundances. In addition, 0.1 was added to both the numerator and the denominator to avoid division by zero.

### Generation of a correlation-based dendrogram

Species present in at least 20% of screening samples from all patients (*n* = 92) were retained, resulting in a total of 306 species. Spearman’s correlation coefficients were calculated between species, and distances were defined as (1—Spearman’s correlation coefficient). Hierarchical clustering with the complete linkage method was then applied to group the species, and a dendrogram was generated.

### TMB calculation from WES of tumor and blood samples

Whole-exome sequencing (WES) data were obtained from tumor and matched blood samples collected at screening (*n* = 70). The Agilent SureSelect Target Enrichment protocol was used to prepare standard exome capture libraries. In all cases, the SureSelect Human All Exon V6 probe set was used. Genomic DNA (1 μg) was diluted in EB Buffer and sheared to a target peak size of 150–200 bp using the LE220 Focused-ultrasonicator (Covaris), according to the manufacturer's recommendations. Fragmentation was followed by end repair and the addition of an ‘A’ tail. Agilent adapters were then ligated to the fragments. Once ligation was assessed, the adapter-ligated product was PCR amplified. For exome capture, libraries were mixed with hybridization buffers, blocking mixes, RNase block, and the SureSelect all exon capture library. Hybridization to the capture baits was conducted at 65 °C with the thermal cycler lid heated to 105 °C for 24 h. The captured libraries were then washed and amplified. Final libraries were sequenced on an Illumina NovaSeq system at Macrogen.

Raw reads were quality checked and trimmed using FastQC (v0.11.9) and Trimmomatic (v0.39) [40]. The trimmed reads were then aligned to the human reference genome GRCh38 using BWA (v0.7.17) [41], and BAM files were generated with SAMtools (v1.9) [20]. Duplicates were marked, followed by base quality score recalibration in GATK (v4.3.0.0) [42]. Somatic variants were identified by comparing tumor samples to their matched blood samples with MuTect2 [43], and the classification and impact of these variants were annotated using Funcotator [42]. Finally, TMB was calculated on a per-megabase basis with pyTMB [44], focusing on non-synonymous mutations. Samples with a TMB of more than 300 mut/Mb (*n* = 2) were filtered out.

### Score calculation from RNA-seq of tumor samples

Bulk RNA sequencing (RNA-seq) was performed on tumor samples collected at screening (*n* = 92). Total RNA (100 ng) was used to prepare libraries with the Agilent SureSelect RNA Direct kit, following the manufacturer’s instructions. For exome capture, the Agilent SureSelect XT Human All Exon V6 + UTRs Kit was used according to the Agilent SureSelect Target Enrichment protocol. Libraries were mixed with hybridization buffers, blocking mixes, RNase block, and the SureSelect all exon capture library. Hybridization to the capture baits was conducted at 65 °C with the thermal cycler lid heated to 105 °C for 24 h. The captured libraries were then washed and amplified. Final libraries were sequenced on an Illumina NovaSeq system at Macrogen.

Raw reads were preprocessed to remove low-quality reads and adapter sequences prior to analysis. The processed reads were then aligned to the *Homo sapiens* reference genome (GRCh38) using HISAT2 (v2.1.0) [45]. Transcript assembly of known transcripts was performed using StringTie (v2.1.3b) [46]. Based on the results, transcript and gene expression abundances were calculated for each sample.

Gene set annotations were retrieved from the Molecular Signatures Database (MSigDB), specifically using the Gene Ontology Biological Process gene sets for *Homo sapiens* [47–50]. Only gene sets with at least five genes matching those present in the NSCLC cohort were included in the analysis. Gene set variation analysis (GSVA) was conducted on transcripts per million (TPM) expression data using the GSVA package in R, with a Poisson kernel specified for estimation [51]. This yielded GSVA scores for each sample across all retained gene sets.

Six RNA-seq-derived scores previously linked to ICI outcomes were evaluated, as listed on the ICI Efficacy Information Portal [52]: IFNG [53], expanded IFNG [53], IMPRES [54], IPS [55], TIDE [56], and TLS [57]. IFNG, expanded IFNG, IMPRES, and TLS were calculated using in-house codes with TPM expression data according to the respective scoring methods. TIDE was computed using TIDEpy (https://github.com/jingxinfu/TIDEpy), and IPS was determined using the code provided in the corresponding publication (https://github.com/icbi-lab/Immunophenogram).

### Flow cytometry analysis of PBMC samples

Peripheral blood mononuclear cell (PBMC) samples were collected at screening (*n* = 92) and after C2D1 (*n* = 82) for immune profiling by flow cytometry. PBMCs were isolated by density gradient centrifugation using Ficoll-Paque (GE HealthCare, 17–1440-03) according to the manufacturer’s instructions. After isolation, PBMCs were washed with PBS and centrifuged at 1500 RPM for 3 min at room temperature. Cells were then stained with antibodies for 20 min at 4 °C, washed in FACS buffer (2% FBS in PBS), resuspended in FACS buffer, and analyzed on a FACSLyric instrument (BD Biosciences, Franklin Lakes, NJ). The following antibodies (all from BD Biosciences) were used: anti-CD3 (565,491), anti-CD4 (568,675), anti-CD8 (563,677), anti-CD11b (569,504), anti-CD14 (565,283), anti-CD16 (561,313), anti-CD19 (562,653), anti-CD25 (565,096), anti-CD45 (560,178), anti-CD56 (555,518), anti-CD127 (562,436), anti-LAG3 (565,716), anti-PD-1 (569,465), anti-TIM3 (565,568), and a fixable viability dye (564,406).

Cells were defined using the following gating strategy: CD3^+^ T cells (Live CD45^+^ CD3^+^), CD4^+^ T cells (Live CD45^+^ CD3^+^ CD4^+^), CD8^+^ T cells (Live CD45^+^ CD3^+^ CD8^+^), CD4^+^ LAG3^+^ T cells (Live CD45^+^ CD3^+^ CD4^+^ LAG3^+^), CD4^+^ PD-1^+^ T cells (Live CD45^+^ CD3^+^ CD4^+^ PD-1^+^), CD4^+^ TIM3^+^ T cells (Live CD45^+^ CD3^+^ CD4^+^ TIM3^+^), CD8^+^ LAG3^+^ T cells (Live CD45^+^ CD3^+^ CD8^+^ LAG3^+^), CD8^+^ PD-1^+^ T cells (Live CD45^+^ CD3^+^ CD8^+^ PD-1^+^), CD8^+^ TIM3^+^ T cells (Live CD45^+^ CD3^+^ CD8^+^ TIM3^+^), Tregs (Live CD45^+^ CD3^+^ CD8^−^ CD25^+^ CD127^low^ or Live CD45^+^ CD3^+^ CD4^+^ CD25^+^ CD127^low^), NK cells (Live CD45^+^ CD3^−^ CD56^+^), NKT cells (Live CD45^+^ CD3^+^ CD56^+^), B cells (Live CD45^+^ CD3^−^ CD19^+^), classical monocytes (Live CD45^+^ CD11b^+^ CD14^+^ CD16^−^), intermediate monocytes (Live CD45^+^ CD11b^+^ CD14^+^ CD16^+^), and nonclassical monocytes (Live CD45^+^ CD11b^+^ CD14^−^ CD16^+^).

### Cytokine measurement of plasma samples

Cytokine concentrations in plasma samples collected at screening (*n* = 90) and after C2D1 (*n* = 82) were measured using a bead-based multiplex assay on a Bio-Plex 200 system (Bio-Rad) with Bio-Plex Manager software. Two multiplex panels (both from R&D Systems) were used: the Human Immunotherapy Luminex Performance Assay 25-plex Fixed Panel (LKTM010B) and the Human XL Cytokine Panel (4-plex; LUXLM000B). These panels enabled the quantification of 29 analytes in human plasma samples: CCL2, CCL3, CCL4, CCL5, CCL11, CD40L, CXCL1, CXCL2, CXCL10, GM-CSF, Granzyme B, IFN-α2, IFN-γ, IL-1α, IL-1β, IL-1ra, IL-2, IL-4, IL-6, IL-8, IL-9, IL-10, IL-12 p70, IL-13, IL-15, IL-17A, IL-33, PD-L1, and TNF-α. All controls, samples, and standards were assayed in duplicate, and all incubation steps were performed at room temperature on a horizontal orbital shaker (800 ± 50 rpm), according to the manufacturer’s instructions. Values extrapolated beyond the standard range were included in downstream analyses, while those below the range for which concentrations could not be calculated were imputed as one-fifth of the lowest value for each analyte.

### Statistical analysis and visualization

Survival analyses were performed using Cox proportional hazards regression models and the log-rank test. Additional statistical tests, including Fisher’s exact test, linear mixed-effects models, meta-analysis, PERMANOVA, Spearman’s correlation coefficient, the t-test, and the Wilcoxon rank-sum test, were conducted as appropriate. All statistical analyses and visualizations were carried out using R or Python. SIAMCAT was employed for the comparative analysis of microbial features between groups [58]. Heatmaps were created in R using either the ComplexHeatmap package [59] or the circlize package [60]. The color coding in several figures followed a previously established palette [61].

## Results

### Cohort characteristics

A total of 92 patients were included in the analysis, with 68 receiving combination therapy with pembrolizumab plus chemotherapy (Combi) and 24 receiving pembrolizumab monotherapy (Mono) (Additional file 1: Fig. S1A and Additional file 2: Table S1). Stool, blood, and tumor samples, along with food frequency questionnaires (FFQs), were collected at up to three time points: at screening, after Cycle 2 Day 1 (C2D1), and at progression (Additional file 1: Fig. S1B). Cohort characteristics are summarized in Table 1. In all treated patients, PD-L1 expression of 50% or more accounted for 34.8%, adenocarcinoma 64.1%, liver metastasis 18.5%, and brain metastasis 34.8%. The combination therapy group included 37 responders (54.4%), while the monotherapy group included 13 responders (54.2%).

**Table 1:** Patient characteristics

Characteristics	All (*n* = 92)	Pembrolizumab plus chemotherapy (*n* = 68)	Pembrolizumab (*n* = 24)
Age (yr) at screening, mean ± SD	67.0 ± 9.8	65.9 ± 10.2	70.2 ± 8.1
BMI at screening, mean ± SD	22.9 ± 3.3	22.9 ± 3.6	22.7 ± 2.5
Sex, *n* (%)			
Female	9 (9.8%)	8 (11.8%)	1 (4.2%)
Male	83 (90.2%)	60 (88.2%)	23 (95.8%)
Probiotic use at screening, *n* (%)			
Yes	18 (19.6%)	14 (20.6%)	4 (16.7%)
No	74 (80.4%)	54 (79.4%)	20 (83.3%)
Antibiotic use at screening, *n* (%)			
Yes	39 (42.4%)	32 (47.1%)	7 (29.2%)
No	53 (57.6%)	36 (52.9%)	17 (70.8%)
Smoking status, *n* (%)			
Current	22 (23.9%)	17 (25.0%)	5 (20.8%)
Former	61 (66.3%)	43 (63.2%)	18 (75.0%)
Never	9 (9.8%)	8 (11.8%)	1 (4.2%)
PD-L1 expression, *n* (%)			
< 1%	24 (26.1%)	24 (35.3%)	0 (0.0%)
1–49%	36 (39.1%)	36 (52.9%)	0 (0.0%)
≥ 50%	32 (34.8%)	8 (11.8%)	24 (100.0%)
Histology, *n* (%)			
Adenocarcinoma (ADC)	59 (64.1%)	42 (61.8%)	17 (70.8%)
Squamous cell carcinoma (SCC)	30 (32.6%)	25 (36.8%)	5 (20.8%)
Others	3 (3.3%)	1 (1.5%)	2 (8.3%)
Liver metastasis, *n* (%)			
Yes	17 (18.5%)	13 (19.1%)	4 (16.7%)
No	75 (81.5%)	55 (80.9%)	20 (83.3%)
Brain metastasis, *n* (%)			
Yes	32 (34.8%)	26 (38.2%)	6 (25.0%)
No	60 (65.2%)	42 (61.8%)	18 (75.0%)
Immunotherapy-related adverse event, *n* (%)			
Yes	34 (37.0%)	21 (30.9%)	13 (54.2%)
No	58 (63.0%)	47 (69.1%)	11 (45.8%)
Chemotherapy-related adverse event, *n* (%)			
Yes	22 (23.9%)	22 (32.4%)	0 (0.0%)
No	46 (50.0%)	46 (67.6%)	0 (0.0%)
RECIST v1.1 response, *n* (%)			
Partial response	31 (33.7%)	21 (30.9%)	10 (41.7%)
Stable disease	41 (44.6%)	32 (47.1%)	9 (37.5%)
Progressive disease	20 (21.7%)	15 (22.1%)	5 (20.8%)
Clinical response, *n* (%)			
Responder	50 (54.3%)	37 (54.4%)	13 (54.2%)
Non-responder	42 (45.7%)	31 (45.6%)	11 (45.8%)

Clinical features were compared between responders and non-responders (Additional file 2: Table S2). In the combination therapy group, a statistically significant difference in age was observed, with responders having a lower median age than non-responders (64 versus 69 years; *P* = 0.013). Responders were also less likely to have liver metastases (8.1% versus 32.3%; *P* = 0.015). In the monotherapy group, similar trends in age (68 versus 72 years; *P* = 0.076) and liver metastasis (7.7% versus 27.3%; *P* = 0.300) were observed, although these differences were not statistically significant. Other clinical features, including sex, probiotic use, antibiotic use, smoking status, PD-L1 expression, histology, and brain metastases, showed no significant differences between responders and non-responders in either group.

Nutrient intake was also compared between responders and non-responders (Additional file 2: Table S3). In the combination therapy group, responders had significantly higher intakes of energy (median 2,197.1 versus 1,819.1 kcal; *P* = 0.016), saturated fat (12.4 versus 8.6 g; *P* = 0.049), monounsaturated fatty acids (13.6 versus 9.2 g; *P* = 0.047), carbohydrates (342.2 versus 286.7 g; *P* = 0.007), and thiamine (2.0 versus 1.5 mg; *P* = 0.040). In contrast, responders showed significantly lower intakes of fat (29.6 versus 46.4 g; *P* = 0.021), monounsaturated fatty acids (8.1 versus 14.7 g; *P* = 0.040), polyunsaturated fatty acids (6.0 versus 12.9 g; *P* = 0.009), omega-3 fatty acids (0.8 versus 1.6 g; *P* = 0.021), and omega-6 fatty acids (5.5 versus 11.8 g; *P* = 0.009) in the monotherapy group.

### Identifying response-associated microbial features

**Fig. 1:**
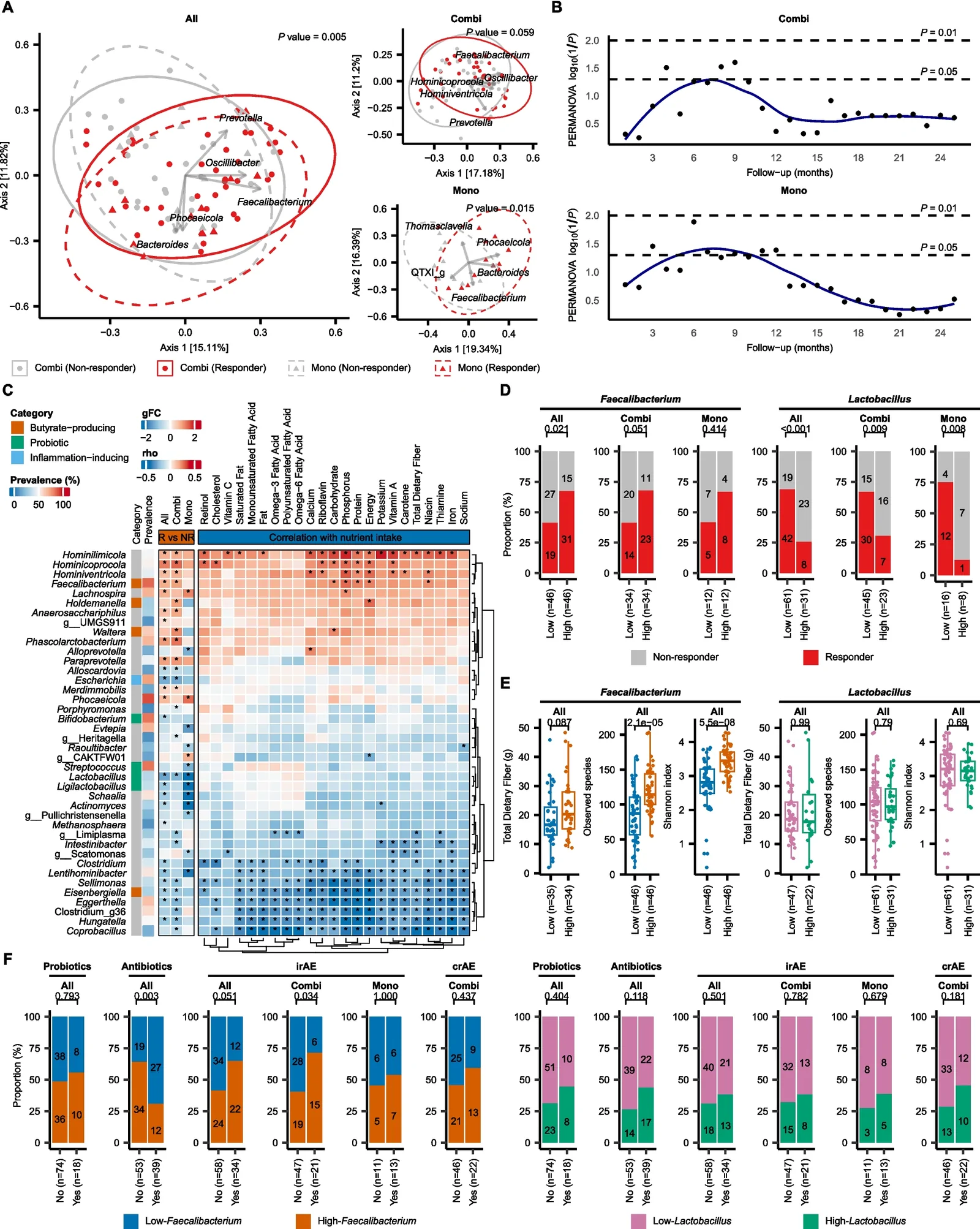
Association between the gut microbiome and response in the Korean NSCLC cohort using screening samples. **A** Principal coordinate analysis (PCoA) was performed based on Bray–Curtis dissimilarity at the genus level. Statistical significance was assessed using permutational multivariate analysis of variance (PERMANOVA) to compare groups stratified by treatment response and type of treatment. **B** The significance of differences in the gut microbiome between patients with disease progression and those without progression across time points was assessed using PERMANOVA 1/*P* of Bray–Curtis dissimilarity at the genus level. **C** The heatmap consists of rows corresponding to genus-level markers enriched in responders and non-responders. Markers were defined as those with *P* values < 0.05 from the Wilcoxon rank-sum test and a prevalence of ≥ 10%. The left annotation provides bacterial categories grouped by functional roles and the prevalence of these markers in the cohort. The left heatmap cells show the generalized fold change (gFC) values derived from SIAMCAT, and asterisks indicate the markers. The right heatmap shows Spearman’s correlation coefficients (rho) between markers and nutrient intake, and asterisks indicate statistical significance (*P* value < 0.05) as determined by correlation tests. **D** The proportion of responders in the high-abundance group versus the low-abundance group. High- and low-abundance groups were defined based on the median abundance of each genus. **E** Differences in total dietary fiber intake and alpha diversity between the high- and low-abundance groups. **F** Differences in clinical features, including probiotic use, antibiotic use, irAEs, and crAEs, between the high- and low-abundance groups. Shotgun sequencing data were used, except for the alpha diversity analysis. *P* values above the plots were calculated using the Wilcoxon rank-sum test or Fisher’s exact test

We first assessed whether overall gut microbial composition differed between responders and non-responders by analyzing taxonomic profiles from screening stool samples (Fig. 1A). Significant differences were observed between groups stratified by treatment response and type of treatment (*P* = 0.005). Within the combination therapy group, differences in microbial composition between responders and non-responders were observed but did not reach statistical significance (*P* = 0.059), whereas the monotherapy group showed a significant difference (*P* = 0.015).

To assess whether overall gut microbiome composition differed between treatment groups, we performed PCoA based on Bray–Curtis dissimilarity at the genus level (Additional file 1: Fig. S2). No significant difference was observed at screening (*P* = 0.190), whereas a trend toward compositional divergence between the two groups emerged after C2D1 (*P* = 0.063).

To identify when the gut microbiome most strongly correlated with clinical outcomes, we compared patients with and without disease progression at various time points after treatment (Fig. 1B). The most pronounced differences in microbial composition were observed 6–9 months after treatment with pembrolizumab, which aligns with the response definition in this cohort. All responders achieved progression-free survival (PFS) of at least 6 months, while all non-responders progressed less than 6 months (Additional file 2: Table S1). This time range differs slightly from a previous report, which identified 9–10 months as the strongest association between baseline microbial composition and clinical outcomes [11], likely due to differences of baseline characteristics. Despite these compositional differences, alpha diversity did not significantly differ between responders and non-responders (Additional file 1: Fig. S3).

We evaluated the association between the gut microbiome and response using a random forest model (Additional file 1: Fig. S4). In both treatment groups, microbial profiles showed modest ability to distinguish responders from non-responders, with area under the receiver operating characteristic curve (AUC) values of 0.61. Interestingly, models trained on monotherapy data were able to classify outcomes in the combination therapy group (AUC = 0.66), and vice versa (AUC = 0.71), suggesting shared microbial markers of response to ICI across treatment types.

To identify microbial features associated with response to pembrolizumab, we compared genus-level taxonomic profiles of baseline stool samples between responders and non-responders (Fig. 1C). Butyrate-producing bacteria such as *Faecalibacterium* were enriched in responders, whereas probiotic bacteria like *Lactobacillus* and inflammation-inducing bacteria such as *Escherichia* were more abundant in non-responders (Additional file 2: Table S4). *Faecalibacterium* remained enriched in responders and *Lactobacillus* in non-responders after C2D1, but these differences were not statistically significant at progression (Additional file 1: Fig. S5).

Next, we investigated *Faecalibacterium* and *Lactobacillus* at baseline stool samples, given their contrasting associations with clinical outcomes. *Faecalibacterium* was enriched in responders and exhibited high prevalence in the NSCLC cohort, whereas *Lactobacillus* was enriched in non-responders across both treatment groups (Fig. 1C). Patients were stratified by the median abundance of each genus: 6.18% for *Faecalibacterium* and 0% for *Lactobacillus* (Additional file 1: Fig. S6). A higher proportion of responders was observed in the *Faecalibacterium*-high group compared to the low group, while the opposite was seen for *Lactobacillus* (Fig. 1D). In addition, patients with high *Faecalibacterium* abundance exhibited longer PFS (median 10.3 versus 4.5 months, *P* = 0.054) and overall survival (OS) (median 21.1 versus 10.4 months, *P* = 0.017), whereas high *Lactobacillus* abundance was associated with shorter PFS (median 3.3 versus 10.7 months, *P* = 0.001) and OS (median 5.1 versus 18.0 months, *P* < 0.001) (Additional file 1: Fig. S7). Notably, high *Faecalibacterium* levels correlated with significantly prolonged OS in the combination therapy group, while high *Lactobacillus* levels were linked to significantly shorter PFS and OS across both treatment groups. Multivariable analysis also showed that high *Faecalibacterium* abundance was associated with improved OS in the combination therapy group, while high *Lactobacillus* abundance was linked to worse PFS and OS in the monotherapy group (Additional file 2: Table S5).

### Microbial associations with dietary, clinical, and host features

We assessed correlations between the genus-level markers and nutrient intake (Fig. 1C). Responder-enriched genera, including *Faecalibacterium*, *Hominicoprocola*, *Hominilimicola*, and *Hominiventricola*, were positively correlated with various nutrient intakes. In contrast, non-responder-enriched genera, including Clostridium_g36, *Coprobacillus*, *Eggerthella*, *Eisenbergiella*, *Hungatella*, and *Sellimonas*, were negatively correlated with many nutrients. Notably, genera such as *Lactobacillus* and *Escherichia* showed no significant correlation with the intake of any nutrient. Similar patterns were observed at the species level (Additional file 1: Fig. S8).

Patients with high *Faecalibacterium* abundance had a higher intake of total dietary fiber (*P* = 0.087), along with other nutrients. In contrast, high *Lactobacillus* abundance was not associated with significant differences in nutrient intake, including total dietary fiber (*P* = 0.995) (Fig. 1E and Additional file 2: Table S6). Alpha diversity was significantly higher in patients with high *Faecalibacterium* abundance, but there were no significant differences of alpha diversity between patients with low and high *Lactobacillus* abundance (Fig. 1E). Clinical features were also compared between the high- and low-abundance groups (Fig. 1F). Antibiotic use was associated with low *Faecalibacterium* abundance (*P* = 0.003) and high *Lactobacillus* abundance (*P* = 0.118). The occurrence of immune-related adverse events (irAEs) was associated with high *Faecalibacterium* abundance in the combination therapy group (*P* = 0.034). Probiotic use and the occurrence of chemotherapy-related adverse events (crAEs) showed no associations with the abundances of *Faecalibacterium* or *Lactobacillus*.

We further analyzed associations between response to ICI and bacterial categories grouped by functional roles of the microbiome, including butyrate-producing, probiotic, and inflammation-inducing bacteria, which are associated with *Faecalibacterium*, *Lactobacillus*, and *Escherichia*, respectively (Additional file 1: Fig. S9 and Additional file 2: Table S4). Butyrate-producing bacteria showed patterns similar to *Faecalibacterium* and was associated with a higher proportion of responders, higher alpha diversity, absence of antibiotic use, and the occurrence of irAEs in the combination therapy group. Likewise, probiotic bacteria showed patterns similar to *Lactobacillus* and was associated with a lower proportion of responders. Inflammation-inducing bacteria showed no significant differences between high- and low-abundance groups.

We assessed how microbial features were associated with host features. Notably, both the *Faecalibacterium*-high and *Lactobacillus*-low groups exhibited enrichment of type I interferon-related gene sets and genes (Additional file 1: Fig. S10), which were induced by the microbiota and associated with improved efficacy of ICI in a previous study [62]. Responder-enriched genus-level markers were negatively correlated with CD4⁺ LAG3⁺, CD8⁺ LAG3⁺, CD4⁺ TIM3⁺, and CD8⁺ TIM3⁺ T cells, as measured by flow cytometry, as well as with multiple pro-inflammatory cytokines, as measured by cytokine assay, including CCL3, CCL4, CCL5, CCL11, CXCL1, CXCL2, CXCL10, GM-CSF, IL-1α, IL-15, and IL-8, suggesting an inverse association with a heightened systemic pro-inflammatory state (Additional file 1: Fig. S11).

### Identifying response-associated microbial gene clusters and major contributing bacteria

**Fig. 2:**
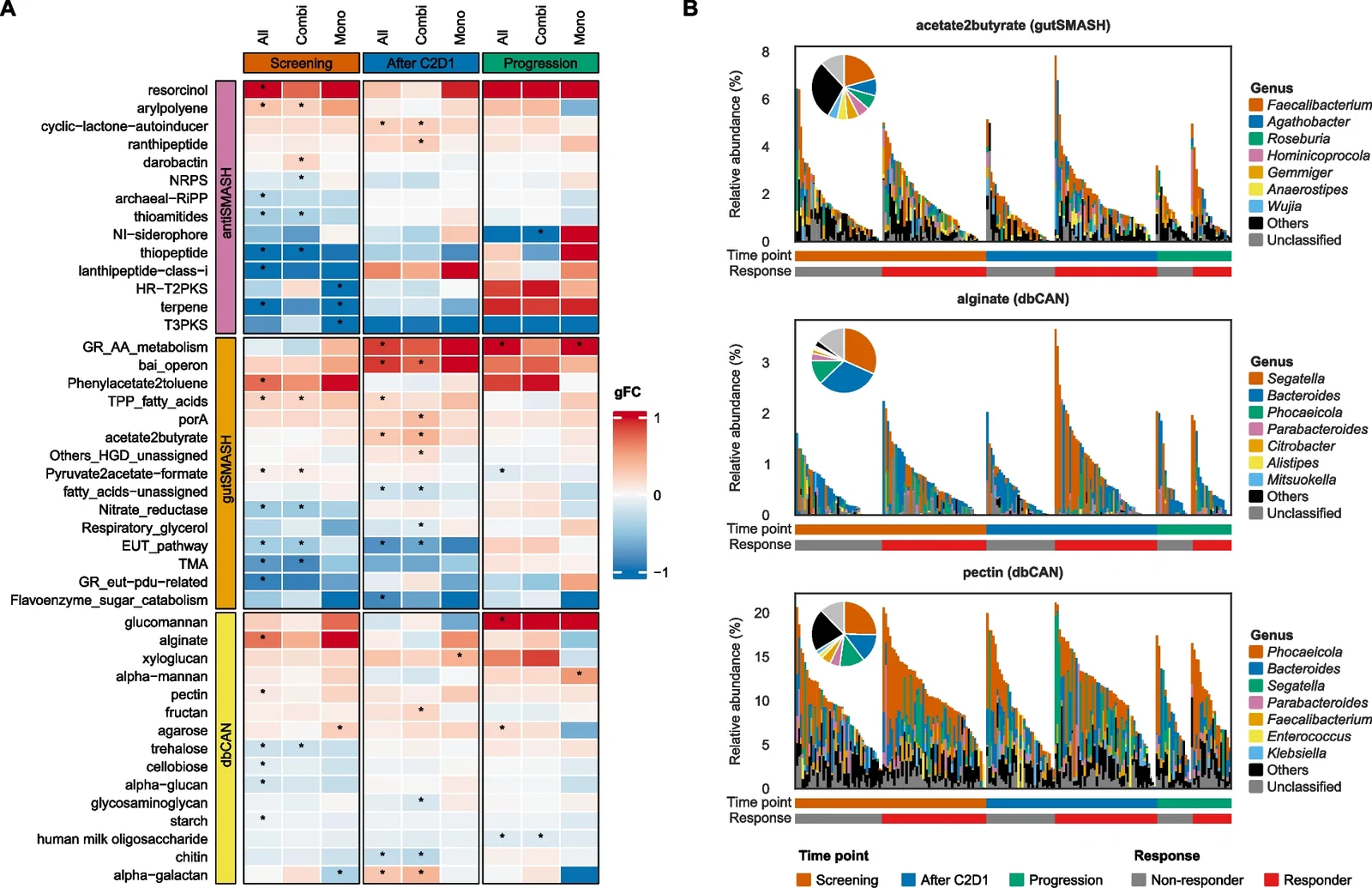
Microbial gene clusters distinguishing responders from non-responders. Gene clusters were detected using antiSMASH, gutSMASH, and dbCAN after metagenomic assembly. **A** The heatmap displays the gFC values derived from SIAMCAT. Asterisks indicate statistical significance (*P* value < 0.05), as determined by the Wilcoxon rank-sum test. Rows with at least one statistically significant result were retained. **B** Bar charts illustrate the relative abundance of specific gene clusters, stratified by contributing genera. Each bar represents a single sample, grouped by time point and response status. Within each group, the bars are arranged in order of total abundance. The pie chart in the upper left corner represents the proportion of contributing genera across all samples

To identify microbial gene clusters associated with response to ICI, we compared those predicted by antiSMASH [34], gutSMASH [30], and dbCAN [35] between responders and non-responders across all time points following metagenomic assembly (Fig. 2A). Gene clusters involved in acetate-to-butyrate biosynthesis, alginate and pectin metabolism, resorcinol biosynthesis, and bile acid dehydroxylation were significantly enriched in responders. In contrast, gene clusters related to ethanolamine utilization, nitrate reduction, trimethylamine (TMA) biosynthesis, and starch and trehalose metabolism were more abundant in non-responders (Fig. 2B and Additional file 1: Fig. S12).

We also identified the major bacterial contributors to each gene cluster (Fig. 2B and Additional file 1: Fig. S12). We found that the acetate-to-butyrate biosynthesis cluster was predominantly found in butyrate-producing bacteria such as *Faecalibacterium*, *Agathobacter*, *Roseburia*, *Gemmiger*, and *Anaerostipes* (Additional file 2: Table S4). Gene clusters associated with alginate metabolism were primarily attributed to *Segatella*, while those involved in pectin metabolism and resorcinol biosynthesis were mainly contributed by *Phocaeicola*. Gene clusters linked to bile acid dehydroxylation were largely associated with g__CAG-103. In contrast, clusters enriched in non-responders were mainly derived from inflammation-inducing bacteria, including *Escherichia*, *Klebsiella*, *Citrobacter*, *Enterobacter*, and *Raoultella* (Additional file 2: Table S4).

### Dynamics of gut microbial signatures in relation to response to ICI

We compared our patient cohort with the 92 healthy subjects in Korea matched for age, BMI, and sex (Additional file 2: Table S7). The overall gut microbial composition was more similar between responders and healthy subjects than between non-responders and healthy subjects (Additional file 1: Fig. S13A). We also calculated the GMI for each sample, which was previously developed to reflect gut microbial health status by incorporating microbial diversity and abundances of commensals and pathobionts [28]. The GMI, alpha diversity, and abundances of *Faecalibacterium* and butyrate-producing bacteria increased stepwise from non-responders to responders and were most enriched in healthy subjects. In contrast, the abundances of *Lactobacillus*, probiotic bacteria, and inflammation-inducing bacteria declined in the same order, highlighting that responders had gut microbial characteristics much closer to those of healthy subjects than did non-responders (Additional file 1: Fig. S13B).

**Fig. 3:**
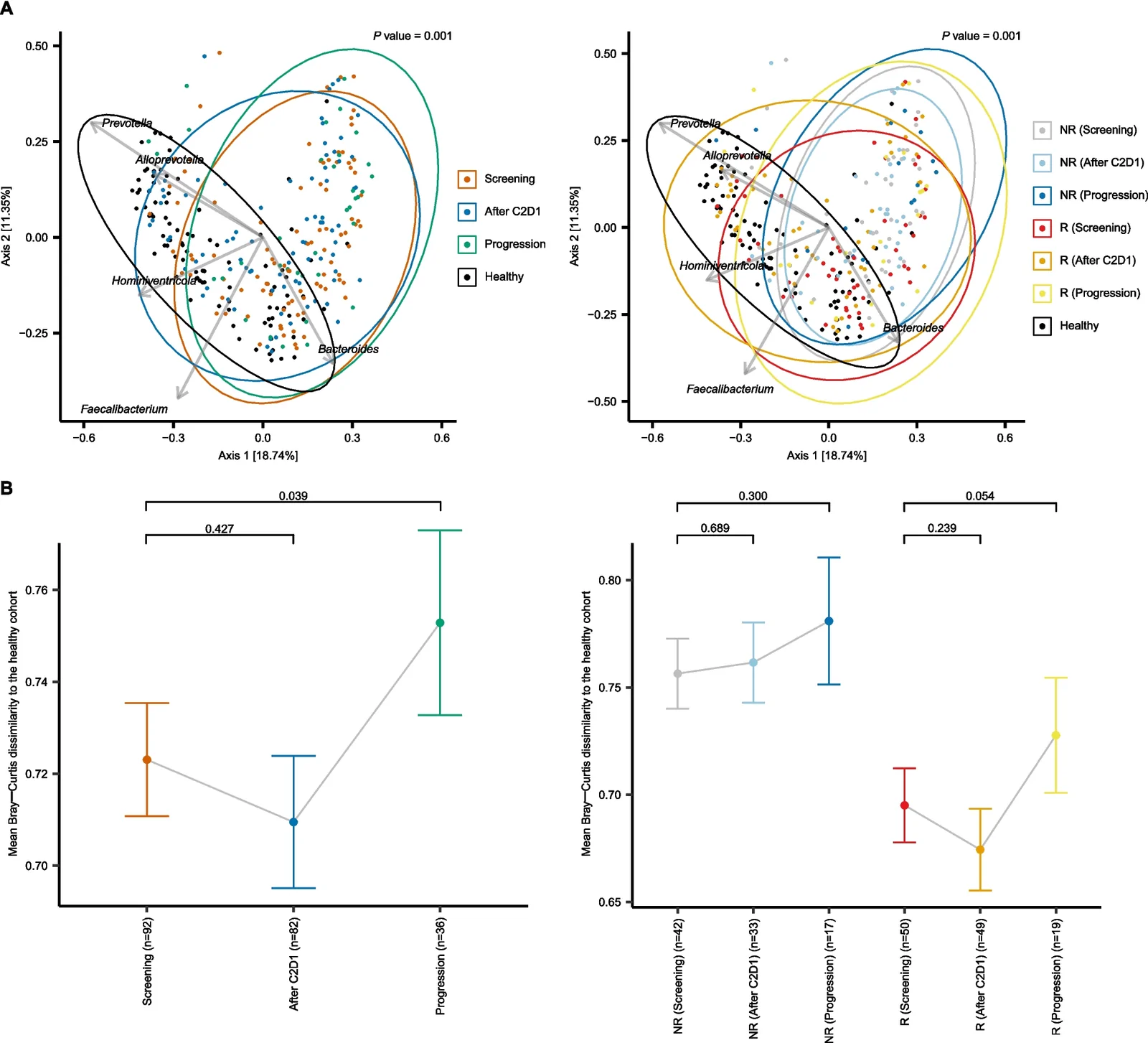
Comparison of samples collected at different time points with those from the healthy Korean cohort. **A** PCoA was performed based on Bray–Curtis dissimilarity at the genus level. Statistical significance was assessed using PERMANOVA to compare groups stratified by time point and response status. **B** For each sample in the NSCLC cohort, the mean Bray–Curtis dissimilarity to the healthy cohort was calculated, and the mean ± standard error was plotted for each group. *P* values shown above the plots were obtained from linear mixed-effects models, with time point as a fixed effect and patient as a random effect. R indicates responders, whereas NR indicates non‑responders

Analysis of longitudinal samples revealed temporal changes in the gut microbiome of the patient cohort. We compared samples collected at different time points with those from healthy subjects in Korea (Fig. 3). The Bray–Curtis dissimilarity to the healthy subjects was significantly higher at progression than at screening (*P* = 0.039). Notably, the dissimilarity of responders decreased after C2D1 but increased at progression, while that of non-responders gradually increased after treatment. At progression, the alpha diversity, GMI, and the abundance of *Faecalibacterium* decreased, while the abundances of *Lactobacillus* and *Escherichia* increased (Additional file 1: Fig. S14). In the linear mixed-effects model, with time point and response as fixed effects and patient as a random effect, responders showed a significantly greater increase in the GMI after C2D1 compared to baseline, relative to non-responders in the combination therapy group (*P* = 0.045) (Additional file 1: Fig. S15).

### Cross-cohort bacterial ratio score associated with response to ICI

We next conducted an integrated analysis of our NSCLC cohort alongside nine publicly available ICI-treated cohorts [5, 6, 10, 11, 36–39] to investigate shared microbial associations. After quality control (Additional file 1: Fig. S16), 511 samples from six discovery cohorts and 217 samples from three validation cohorts were retained for analysis (Additional file 2: Tables S8 and S9).

**Fig. 4:**
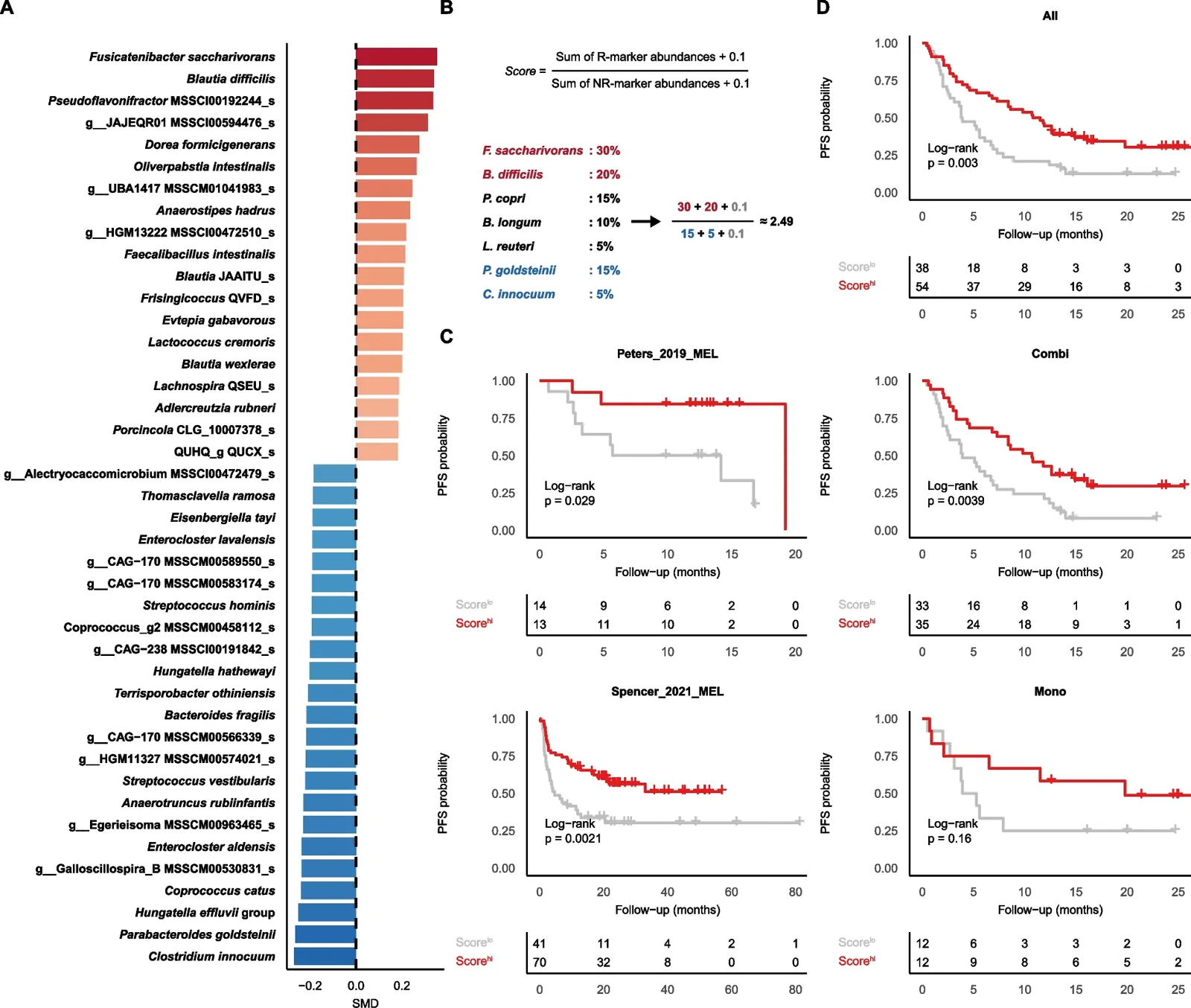
Meta-analysis of the gut microbiome in publicly available cohorts and its application to the validation and Korean cohorts. **A** Species-level markers distinguishing responders from non-responders in the discovery cohorts. Statistical significance was assessed using a random-effects meta-analysis, and markers with *P* values < 0.05 that were found in all six cohorts were retained. SMD, standardized mean difference. **B** Calculation method for the bacterial ratio score and example. R indicates responders, whereas NR indicates non-responders. Survival curves for patients with high and low bacterial ratio scores in the validation cohorts (**C**) and in the Korean NSCLC cohort (**D**). High and low groups were defined using the optimal threshold based on Youden’s index, and only first-visit samples were used. *P* values were calculated using the log-rank test

To identify microbial features associated with response in the public datasets, we performed a random-effects meta-analysis across the six discovery cohorts (Fig. 4A), identifying 19 species enriched in responders and 23 in non-responders. Notably, responder-enriched species consistently showed significantly higher prevalence across all cohorts (Additional file 1: Fig. S17).

To evaluate the relevance of these microbial features to treatment outcomes and identify shared associations across the Korean and publicly available cohorts, we developed a response-associated bacterial ratio score. This score is defined as the ratio of the total abundance of responder-enriched species to that of non-responder-enriched species (Fig. 4B). To minimize potential cohort-specific bias, microbial features identified through the meta-analysis of public datasets were prioritized over markers from the Korean cohort in developing the score. Applying this score to the discovery cohorts yielded AUC values ranging from 0.56 to 0.84 (Additional file 1: Fig. S18).

We then applied the bacterial ratio score to the three validation cohorts and our NSCLC cohort, observing AUC values between 0.59 and 0.69, except for Matson_2018_MEL, which yielded an AUC of 0.44 (Additional file 1: Fig. S19A–D). Notably, performance on this dataset also failed to exceed random classification (AUC = 0.5) in a previous study [63]. We additionally evaluated an NSCLC-specific score derived from the two public NSCLC cohorts in our NSCLC cohort (Additional file 1: Fig. S20). Patients were stratified based on the optimal threshold that maximized Youden’s index. In the validation cohorts, patients with a high bacterial ratio score had significantly longer PFS (Fig. 4C). In our cohort, a high score was associated with longer PFS and OS, with statistical significance observed in the combination therapy group but not in the monotherapy group (Fig. 4D and Additional file 1: Fig. S19E).

As cohort-specific threshold optimization may inflate apparent performance, we derived a single fixed threshold of 1.656 from the pooled discovery cohorts (*n* = 511) using Youden’s index and applied it unchanged to all validation cohorts and the Korean NSCLC cohort (Additional file 1: Fig. S21A; Additional file 2: Table S10). In the Korean NSCLC cohort, this fixed threshold yielded results consistent with the per-cohort approach: response rate, 69.0% versus 42.0% (*P* = 0.012; per-cohort: 66.7% versus 36.8%, *P* = 0.006); PFS, HR = 0.48 (95% CI 0.29–0.79, *P* = 0.004; per-cohort: HR = 0.49, 95% CI 0.30–0.79, *P* = 0.004); and OS, HR = 0.42 (95% CI 0.24–0.73, *P* = 0.002; per-cohort: HR = 0.44, 95% CI 0.26–0.75, *P* = 0.003).

The Korean NSCLC cohort achieved C-index for PFS = 0.609 and C-index for OS = 0.607 (Additional file 2: Table S10); at AUC = 0.635, the bacterial ratio score outperformed the majority of its 42 component species across cohorts (Additional file 2: Table S11). Response rates across five pre-specified score quintile bins showed a significant positive trend in the Korean NSCLC cohort (Cochran–Armitage *P* = 0.048; Additional file 1: Fig. S21B–C).

To further explore shared microbial associations, we constructed a dendrogram based on species correlations within the NSCLC cohort (Additional file 1: Fig. S22). Although there was limited overlap in the markers between the Korean and publicly available cohorts, they clustered closely, suggesting shared underlying microbial signatures.

We assessed the relationship between the bacterial ratio score and GMI. A significant positive correlation was observed between the bacterial ratio score and GMI (*P* < 0.001) (Additional file 1: Fig. S23A). The GMI was also associated with response, although to a lesser extent than the bacterial ratio score (Additional file 1: Fig. S23B–E). We also combined the bacterial ratio score and GMI for subsequent analyses. Patients were stratified according to the optimal cutoffs for GMI and the bacterial ratio score, which were determined using Youden's index. Patients with both high GMI and high bacterial ratio scores had longer PFS and OS, as well as a higher proportion of responders (Additional file 1: Fig. S24). In contrast, patients with both low GMI and low bacterial ratio scores had shorter PFS and OS, as well as a lower proportion of responders (Additional file 1: Fig. S24). Response classification using both GMI and the bacterial ratio score yielded a numerically higher AUC than classification using either metric alone in the Korean NSCLC cohort, though this improvement was not observed in the monotherapy group (Additional file 1: Fig. S25).

### Integrative stratification of response to ICI using host and microbial features

**Fig. 5:**
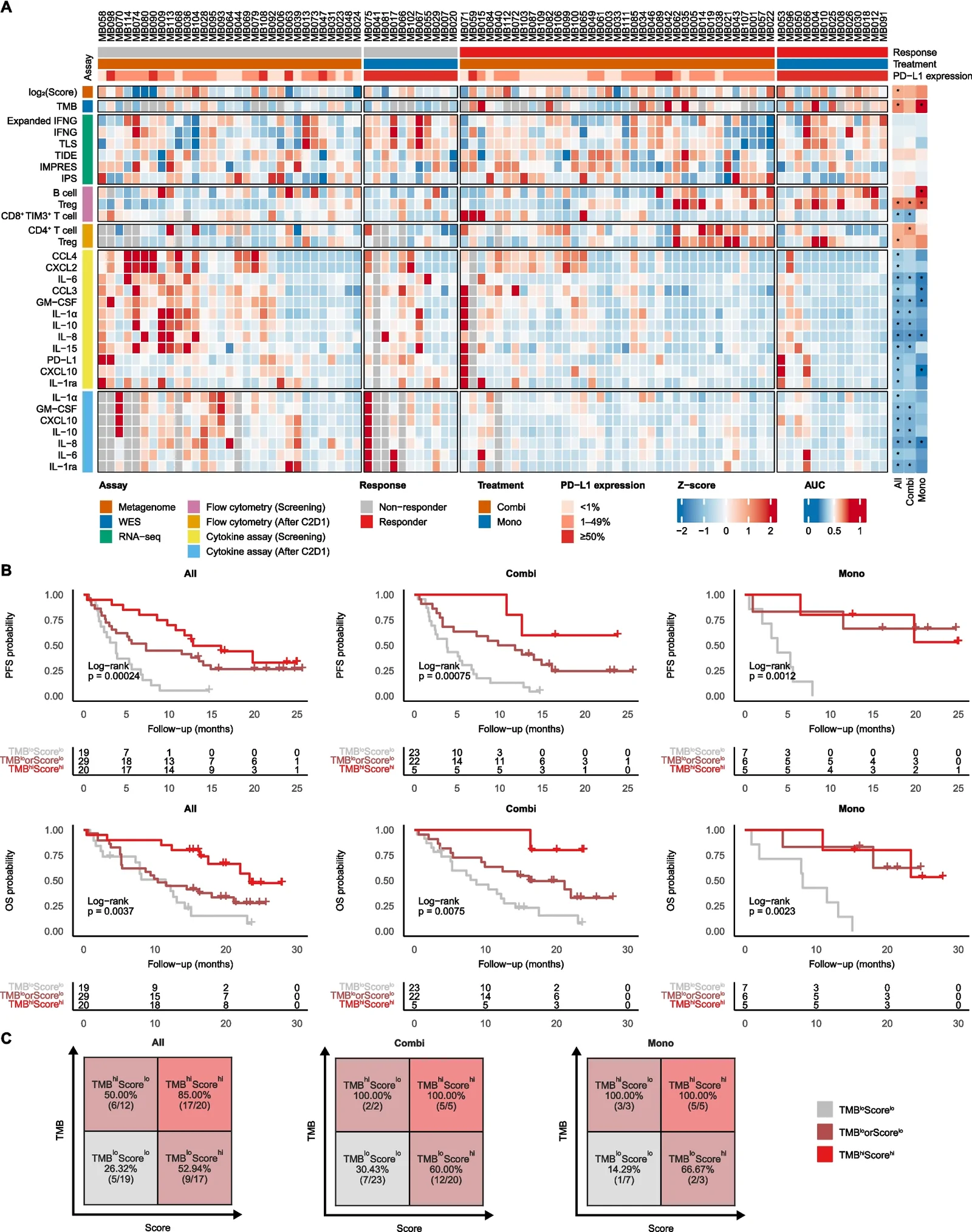
Utilization of multi-omics data to stratify response to ICI in the Korean NSCLC cohort. **A** Heatmap of selected features across individual patients. The top annotation provides patient information, including response status, treatment type, and PD-L1 expression, while the left annotation indicates the assay from which each feature was derived. Rows correspond to features including the log2-transformed bacterial ratio score, TMB, RNA-seq-derived scores (IFNG, expanded IFNG, IMPRES, IPS, TIDE, and TLS), and features derived from flow cytometry and cytokine assays that significantly differ between responders and non-responders. Each heatmap cell shows the Z-score normalized value of the corresponding feature. The panel on the right displays the AUC for each feature in distinguishing responders from non-responders. Asterisks indicate statistical significance (*P* value < 0.05), as determined by the Wilcoxon rank-sum test. **B** Survival curves comparing patients grouped by combined TMB and the bacterial ratio score: both high, both low, or discordant (high/low or low/high) in the Korean NSCLC cohort. High and low groups were defined using the optimal threshold based on Youden’s index, with only first-visit samples included. *P* values were calculated via the log-rank test. **C** Proportion of responders in each group stratified by combined TMB and the bacterial ratio score

We next integrated host and microbial features to stratify treatment response. We first assessed the associations between various features from different assays and response to ICI in the NSCLC cohort (Fig. 5A). TMB, calculated from WES data, showed no correlation with the bacterial ratio score (*P* = 0.459) (Additional file 1: Fig. S26A), but was associated with improved response in this cohort (Additional file 1: Fig. S26B–E).

We also calculated several transcriptomic scores previously linked to ICI outcomes, including IFNG [53], expanded IFNG [53], IMPRES [54], IPS [55], TIDE [56], and TLS [57]; however, none of these scores exceeded AUC values of 0.6 (Additional file 2: Table S12).

We compared features from flow cytometry and cytokine assays between responders and non-responders (Fig. 5A). Notably, B cells and Tregs were enriched in responders, while CD8^+^ TIM3^+^ T cells were enriched in non-responders at screening. Additionally, CD4^+^ T cells and Tregs were enriched in responders after C2D1. In the cytokine assay, several pro-inflammatory cytokines, including CXCL10, GM-CSF, IL-1α, and IL-8, were enriched in non-responders at both time points, suggesting a heightened systemic pro-inflammatory state in non-responders.

We integrated both TMB and bacterial ratio score, which were associated with response to pembrolizumab, and patients were stratified based on the optimal thresholds for TMB and the bacterial ratio score that maximized Youden’s index. Patients with high TMB and high bacterial ratio score showed longer PFS and OS (Fig. 5B) and had a higher proportion of responders (Fig. 5C). In contrast, those with low TMB and low bacterial ratio score had shorter PFS and OS (Fig. 5B) and had a lower proportion of responders (Fig. 5C). Response classification using both TMB and bacterial ratio score yielded a higher AUC than using either metric alone (Additional file 1: Fig. S27). Given the lack of correlation between the two (Additional file 1: Fig. S26A), these results suggest that the bacterial ratio score explains a distinct aspect of response to ICI, which is independent of TMB. To assess the independent contribution of these markers, we performed multivariable Cox regression analysis in patients with available TMB data (Additional file 2: Table S13). Both the bacterial ratio score and TMB were independently associated with longer PFS after adjustment for GMI, treatment type, age, BMI, sex, probiotic use, antibiotic use, smoking status, PD-L1 expression, histology, liver metastasis, and brain metastasis (bacterial ratio score: HR = 0.403, 95% CI 0.175–0.929, *P* = 0.033; TMB: HR = 0.461, 95% CI 0.229–0.927, *P* = 0.030). For OS, TMB remained independently associated with longer survival (HR = 0.401, 95% CI 0.187–0.857, *P* = 0.018), while the bacterial ratio score showed a favorable but nonsignificant association (HR = 0.540, 95% CI 0.226–1.286, *P* = 0.164). Together, these results indicate that the bacterial ratio score is independently associated with PFS, although its association with OS requires validation in a larger cohort.

## Discussion

In this study, we identified microbial features associated with response to immunotherapy in patients with advanced NSCLC. Notably, *Faecalibacterium*, as well as butyrate-producing bacteria, was linked to improved response to ICI. *Faecalibacterium* has been reported as a response-associated marker in multiple studies [4, 12, 39] and produces butyrate [64], which is associated with response to ICI [65]. Our study showed that *Faecalibacterium* reflected a healthy gut status, given its association with high alpha diversity and the absence of antibiotic use. Antibiotics, which induce gut dysbiosis [66], are generally associated with a limited response to ICI [5, 67, 68]. *Faecalibacterium* was also associated with high dietary fiber intake, as reported in a previous study [69]. High dietary fiber intake is associated with improved clinical benefit to immunotherapy [37] and is currently being explored as a potential intervention in patients with melanoma receiving immunotherapy [70].

In contrast, *Escherichia*, *Lactobacillus*, and probiotic bacteria were associated with non-response. *Escherichia* was linked to non-response to ICI in patients with melanoma in a previous study [4]. Probiotic use was associated with impaired antitumor response to ICI in preclinical models, while improved therapeutic response to ICI was observed in patients with sufficient intake of dietary fiber and absence of probiotic use in a previous study [37]. However, this previous study did not assess the relationship between probiotic use and the abundance of probiotic bacteria. In our study, no significant association between them was observed. One plausible explanation is that the high abundance of *Lactobacillus* or probiotic bacteria may reflect dysbiosis regardless of probiotic use, given the observed, but non-significant, association with antibiotic use.

Previously, various studies have shown that gut microbiome is associated with therapeutic response to immunotherapies in advanced NSCLC. Diversity of gut microbiome was associated with favorable response to anti-PD-1 agent in a Chinese cohort treated with nivolumab [71]. Alpha diversity also correlated with OS in Japanese patients with advanced NSCLC treated with anti-PD-1/PD-L1 inhibitors [72], while gut microbial composition differed according to PFS, and abundance of *Firmicutes* and *Actinobacteria* phyla was associated with shorter PFS in Caucasian patients treated with anti-PD-1 agent [73]. These findings are broadly consistent with our observations that responders exhibited microbial characteristics associated with healthier gut microbiota status, including enrichment of *Faecalibacterium*, butyrate-producing bacteria, and higher alpha diversity.

We also identified response-associated microbial gene clusters in the NSCLC cohort treated with pembrolizumab. Gene clusters related to alginate and pectin metabolism were enriched in responders, while those related to starch and trehalose metabolism were enriched in non-responders, highlighting the role of dietary fiber in improving treatment outcome. Resorcinol-based compounds are relevant to response to ICI [74]. Similarly, we found that the resorcinol-related gene cluster was enriched in responders. Responders also showed enrichment of the gene cluster related to bile acid dehydroxylation, a process that transforms primary bile acids into secondary bile acids, which are known to inhibit the growth of *Clostridium difficile* [75]. In contrast, gene clusters involved in ethanolamine utilization, nitrate reduction, and TMA biosynthesis were enriched in non-responders. Genes related to ethanolamine utilization have been reported in many gut pathogens [76]. Nitrate, generated as a by-product of the inflammatory response, confers a growth advantage to *Escherichia coli*, a prototypic member of the Enterobacteriaceae that possesses multiple nitrate reductases [77]. TMA is a precursor of trimethylamine N-oxide (TMAO), which can either promote tumor growth [78, 79] or enhance antitumor immunity [80, 81], depending on the cancer type and biological context.

Our study showed that responders to ICIs exhibited gut microbial characteristics more similar to those of healthy subjects than did non-responders. Alpha diversity and GMI increased stepwise in the order of non-responders, responders, and healthy subjects. Distinct microbial dynamics were also observed during treatment with pembrolizumab between responders and non-responders. Dissimilarity from the healthy cohort decreased after C2D1 only in responders but increased at progression in both groups. Collectively, these findings suggest a bidirectional interaction between the gut microbiome and host health, with each potentially influencing the other, and imply that a healthy gut microbiome may be important for an improved response to ICIs, aligning with previous studies showing the efficacy of FMT [7–9]. Characterizing dysbiosis before therapy initiation with parameters such as gut microbial distance from healthy subjects and GMI may help identify patients more likely to respond to ICIs.

We developed the bacterial ratio score based on 19 bacterial species enriched in responders and 23 in non-responders, which was associated with improved PFS in the validation cohorts and with improved PFS and OS in our cohort. Bacterial community markers may be more robust for cross-cohort response stratification than single-taxon markers, given the functional redundancy among bacteria [82] and the nature of the gut microbiome as a complex ecosystem [83]. A previous study also developed a dysbiosis score from response-associated bacterial communities based on the ecological topology of the gut microbiota [12]. Similarly, the species included in the bacterial ratio score displayed ecological clustering in the correlation-based dendrogram. In addition, the score correlated positively with GMI, suggesting that it reflects gut microbial health. Responder-enriched species, which were used in the calculation of the score, were consistently prevalent across cohorts, a pattern that was also observed previously [12], implying that common microbes may indicate a healthier microbial state.

Although the positive correlation between the bacterial ratio score and GMI was statistically significant, its magnitude was modest. The improved response stratification observed when combining the two metrics in the Korean cohort suggests that the bacterial ratio score may capture gut microbial characteristics not fully reflected by GMI. Notably, the non-responder-enriched marker component of the bacterial ratio score includes taxa such as *Enterocloster* and *Clostridium innocuum*, which have recently been reportd to be depleted in responders following FMT [84], suggesting a functional role in ICI resistance. Incorporating these additional microbial features may further improve response classification.

The differential patterns observed between monotherapy and combination groups warrant further exploration. At baseline, beta-diversity separation between responders and non-responders reached significance in the monotherapy (*P* = 0.015) but not in the combination group (*P* = 0.059). In contrast, other microbiome–response associations were evident in the combination group, including significant associations of *Faecalibacterium* abundance and the bacterial ratio score with clinical outcomes, as well as a greater post-C2D1 increase in GMI among responders than non-responders in a linear mixed-effects model (*P* = 0.045). Despite these treatment-specific patterns, random forest models trained on the monotherapy group distinguished responders from non-responders in the combination group (AUC = 0.66), and vice versa (AUC = 0.71). Furthermore, *Lactobacillus* showed consistent associations with worse outcomes in both groups, suggesting that shared microbiome-driven response determinants may operate across both treatment contexts, even as the specific patterns through which these associations are expressed differ by treatment. In addition, direct beta-diversity comparison between the two groups showed comparable overall compositions at screening (*P* = 0.190), whereas a trend toward divergence emerged after C2D1 (*P* = 0.063), suggesting that the addition of chemotherapy may progressively alter gut microbiome composition. This pattern is in line with a previous study showing that gut microbial composition differed significantly between patients who were chemotherapy-naïve and chemotherapy-treated [73]. In our study, all patients were treatment-naïve, and chemotherapy was administered concurrently with pembrolizumab as first-line therapy. However, the imbalance in sample size between groups (combination group *n* = 68; monotherapy group, *n* = 24) and the small sample size in the monotherapy group limit the statistical power for formal subgroup comparisons. Larger, prospectively stratified cohorts with balanced treatment arms in the future will help elucidate the specific contributions of pembrolizumab and chemotherapy.

We integrated host and microbial signatures to assess response to ICI. Comparison of flow cytometry and cytokine assay revealed that Tregs were enriched in responders, while several pro-inflammatory cytokines were elevated in non-responders. This suggests a markedly pro-inflammatory systemic immune state in non-responders, potentially predisposing them to chronic inflammation or immune dysregulation. Microbial markers enriched in responders were also negatively associated with the heightened systemic pro-inflammatory state. Given that non-responders exhibited dysbiotic microbial signatures, the pro-inflammatory immune state may be associated with compromised gut status. Our findings support previous studies that have reported that gut dysbiosis can lead to the uncontrolled translocation of bacterial components, potentially triggering systemic inflammation [85–87].

Although scores derived from RNA-seq data showed limited ability to distinguish responders from non-responders, both TMB and the bacterial ratio score were able to stratify response in this cohort. TMB is actively being studied as a useful biomarker for response to ICI, with high TMB levels generally associated with improved clinical outcomes [88–90]. We integrated TMB with the bacterial ratio score to further enhance response stratification. The independence of these two metrics strengthens the argument that the bacterial ratio score captures biological features distinct from those reflected by TMB, specifically gut microbial status rather than neoantigenicity.

Recent studies have demonstrated that intratumoral bacteria can shape the tumor immune microenvironment across multiple cancer types [91–93]. The present study focused on gut microbiome profiling from stool samples; the interaction between intratumoral bacteria and the tumor immune microenvironment was not examined, as neither intratumoral microbiome characterization nor spatial profiling of paired tissue data was performed. This interaction warrants further investigation in future studies with dedicated tumor tissue profiling.

Our study has several limitations. First, our study was conducted at a single center in a Korean cohort. Considering the heterogeneity of the patient population, dietary habits and ethnicity, our results should be interpreted with caution and require validation in independent cohorts. Second, the sample size was relatively modest and patients received heterogeneous treatment of pembrolizumab monotherapy or in combination with chemotherapy, and not all patients had available tissues for WES. Third, although we adjusted for major clinical variables, the possibility of residual confounding cannot be completely excluded. Finally, the public cohorts included in the meta-analysis were retrospectively collected and differed in sequencing protocols and treatment settings. Despite these limitations, our study provides a comprehensive analysis of host and microbial signatures in advanced NSCLC treated with pembrolizumab in an Asian population.

## Conclusions

In summary, our findings underscore the critical relevance of gut microbial health to the response to ICI. In this cohort, microbial health was reflected in the abundance of specific taxa, such as *Faecalibacterium* and *Lactobacillus*, which showed distinct associations with dietary intake and antibiotic use. Beyond baseline associations, we observed dynamic shifts in microbial signatures throughout treatment and disease progression, further supporting their prognostic and therapeutic relevance. We also developed the bacterial ratio score that was strongly associated with response to ICI and demonstrated robust classification performance across independent cohorts, underscoring its generalizability. This score was negatively associated with dysbiosis and enhanced response stratification when combined with TMB. Notably, the score was independent of TMB, suggesting a distinct and complementary axis of response and emphasizing the utility of multimodal biomarkers. Together, these findings highlight the value of routine monitoring and modulation of gut microbial health as part of cancer care and support the development of integrated biomarker strategies.

## Supplementary Information

Additional file 1 : Supplementary Figures 1–27.

Additional file 2 : Supplementary Tables 1–13.

## Data Availability

The shotgun and amplicon sequencing data from patients with NSCLC and healthy subjects are available in the NCBI Sequence Read Archive (SRA) under BioProject accession numbers PRJNA1268044 [94] and PRJNA1268045 [95], respectively. Shotgun sequencing data from previously published studies are available under the following accession numbers: PRJEB22863, PRJEB43119, PRJNA397906, PRJNA399742, PRJNA541981, PRJNA751792, PRJNA762360, and PRJNA770295 (see Additional file 2: Tables S8 and S9).

## Abbreviations

ADC: Adenocarcinoma
ASV: Amplicon sequence variant
AUC: Area under the receiver operating characteristic curve
C2D1: Cycle 2 Day 1
CAZyme: Carbohydrate-active enzyme
CI: Confidence interval
CLR: Centered log-ratio
Combi: Combination therapy with pembrolizumab plus chemotherapy
crAE: Chemotherapy-related adverse event
CT: Computed tomography
CTCAE: Common Terminology Criteria for Adverse Events
ECOG: Eastern Cooperative Oncology Group
FFPE: Formalin-fixed, paraffin-embedded
FFQ: Food frequency questionnaire
FMT: Fecal microbiota transplantation
gFC: Generalized fold change
GMI: Gut microbiome index
GSVA: Gene set variation analysis
HR: Hazard ratio
ICI: Immune checkpoint inhibitor
irAE: Immune-related adverse event
JSD: Jensen–Shannon distance
KDCA: Korea Disease Control and Prevention Agency
KNHANES: Korea National Health and Nutrition Examination Survey
MAG: Metagenome-assembled genome
MFDS: Ministry of Food and Drug Safety
Mono: Pembrolizumab monotherapy
MSigDB: Molecular Signatures Database
NSCLC: Non-small cell lung cancer
OS: Overall survival
PBMC: Peripheral blood mononuclear cell
PCoA: Principal coordinate analysis
PCR: Polymerase chain reaction
PERMANOVA: Permutational multivariate analysis of variance
PFS: Progression-free survival
RECIST: Response Evaluation Criteria in Solid Tumors
rho: Spearman’s correlation coefficient
RNA-seq: RNA sequencing
ROC: Receiver operating characteristic
SCC: Squamous cell carcinoma
SMD: Standardized mean difference
TMA: Trimethylamine
TMAO: Trimethylamine N-oxide
TMB: Tumor mutational burden
TPM: Transcripts per million
TPS: Tumor proportion score
WES: Whole-exome sequencing
