# Supplementary material for: Integrative stratification of response to immunotherapy using host and microbial signatures in patients with advanced non-small cell lung cancer
Source: Genome Med. 2026 Aug 6;18:170. doi: 10.1186/s13073-026-01736-5 (PMC13729566; doi:10.1186/s13073-026-01736-5)
Supplement: Supplementary file 1 [file 13073_2026_1736_MOESM1_ESM.pdf]

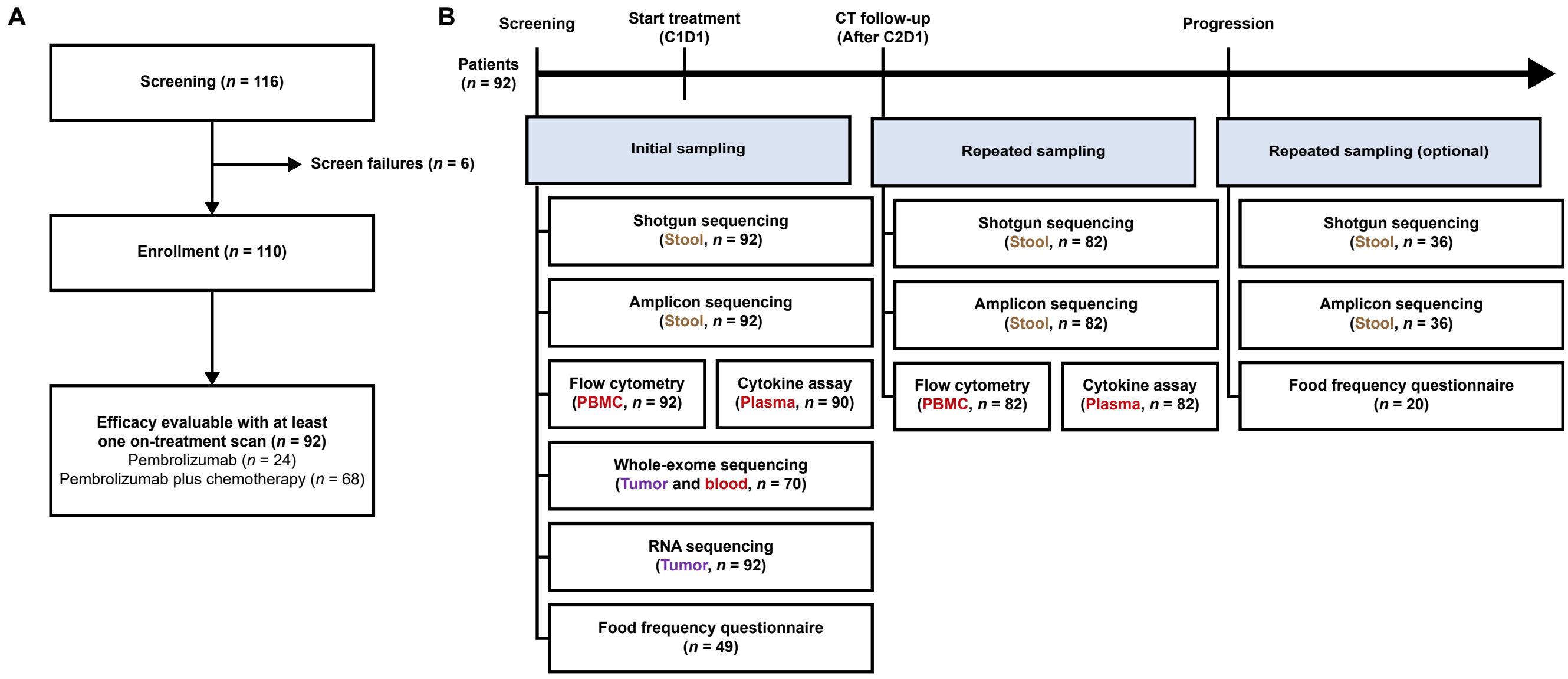

Fig. S1 Sample information from the Korean NSCLC cohort. **A** The flow diagram illustrates patient inclusion. **B** The diagram shows the number of samples for different assays collected at up to three time points: at screening, after C2D1, and at progression.

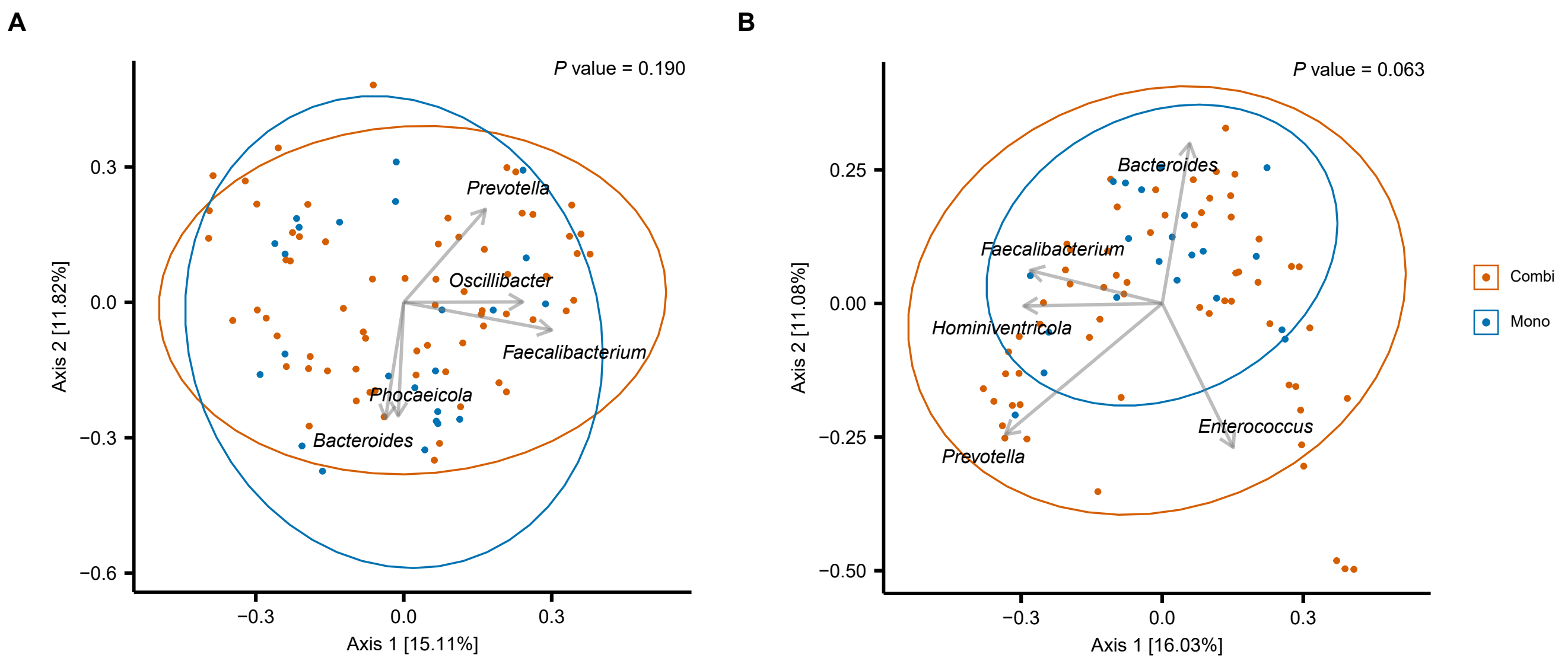

Fig. S2 Principal coordinate analysis (PCoA) was performed based on Bray–Curtis dissimilarity at the genus level. Statistical significance was assessed using permutational multivariate analysis of variance (PERMANOVA) to compare groups stratified by treatment type using samples collected at screening (**A**) and after C2D1 (**B**).

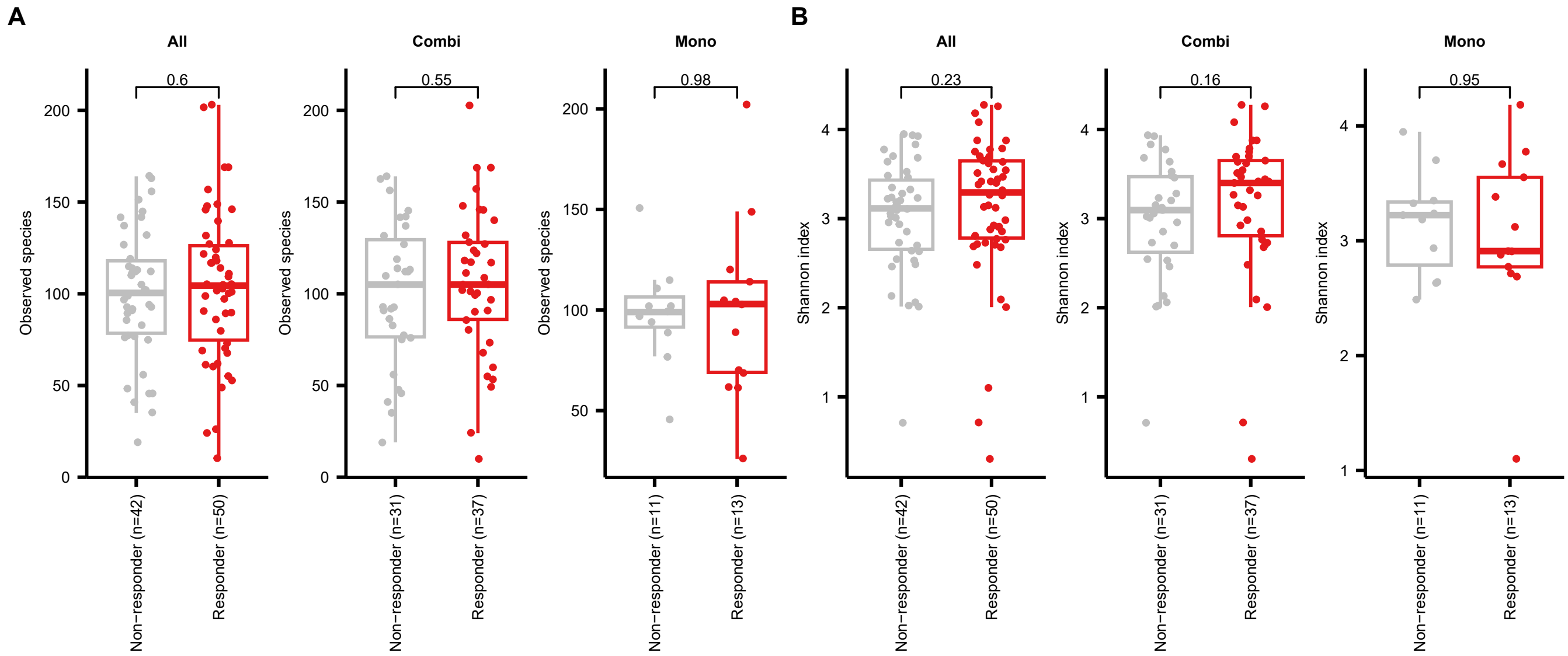

Fig. S3 Alpha diversity evaluated at the species level using the number of observed species and the Shannon index. The number of observed species (**A**) and the Shannon index (**B**) were compared between responders and non-responders. *P* values above the box plots were calculated using the Wilcoxon rank-sum test. Amplicon sequencing data were used, and results are shown for samples collected at screening.

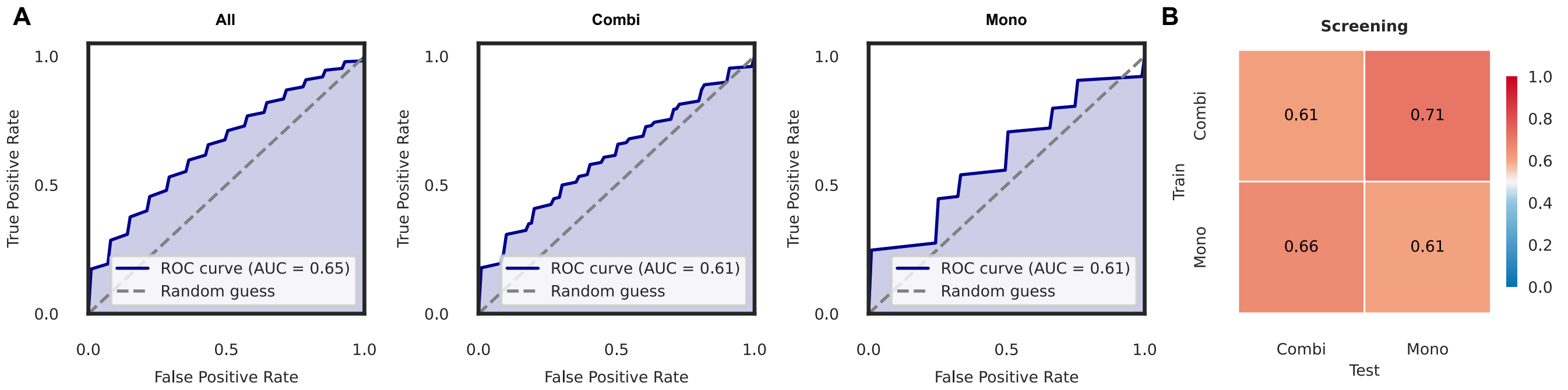

Fig. S4 Response classification using a random forest model with species-level profiles. Receiver operating characteristic (ROC) curves and an AUC matrix were generated using samples collected at screening. **A** ROC curves were calculated from 100-repeated three-fold stratified cross-validation. **B** Diagonal values in the matrix represent the AUC values from 100-repeated three-fold stratified cross-validation. Off-diagonal values indicate AUC values obtained by training the model on the cohort in the corresponding row and applying it to the cohort in the corresponding column, with 100 repetitions.

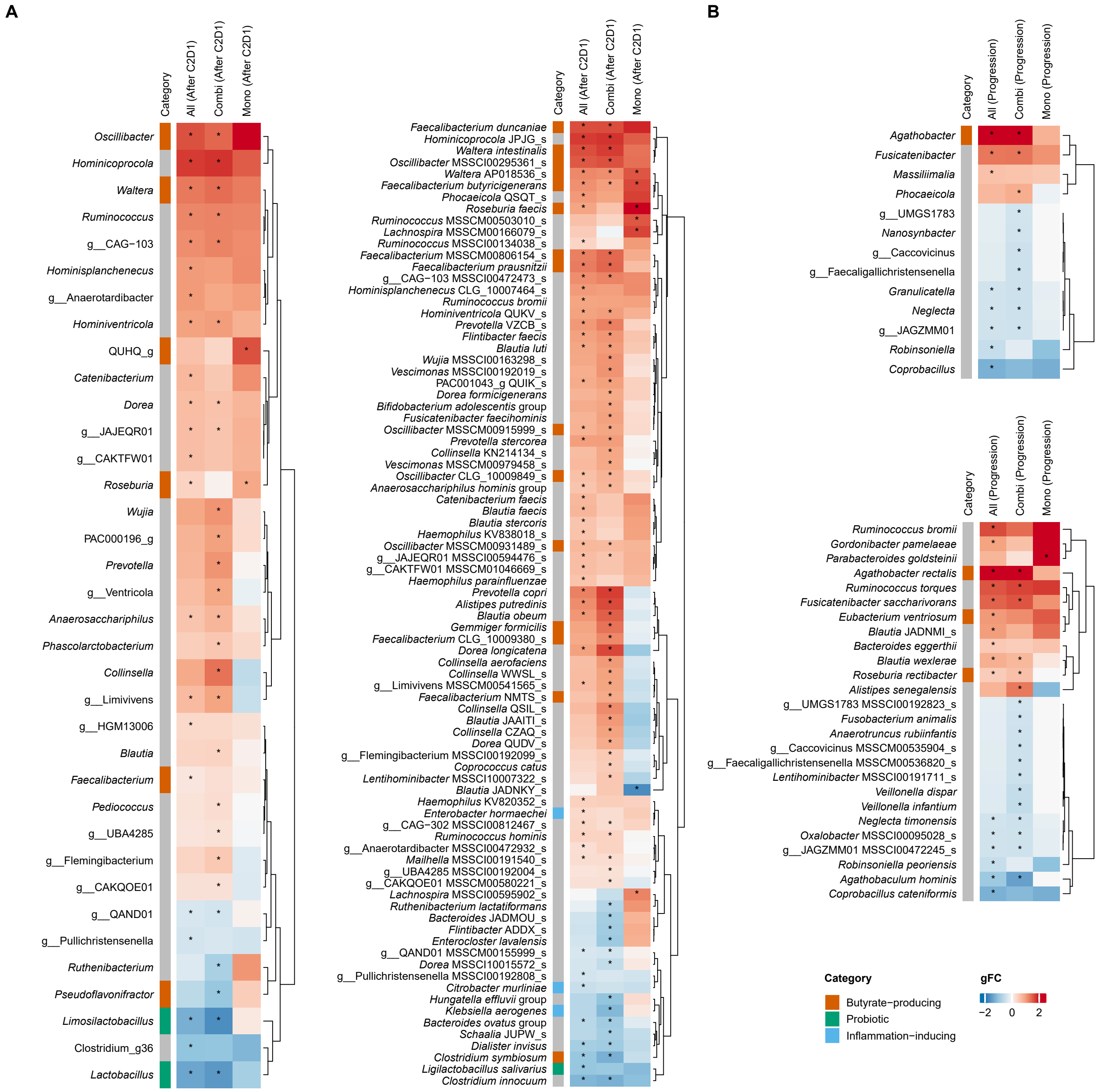

Fig. S5 Taxonomic markers distinguishing responders from non-responders using samples collected after C2D1 and at progression. Genus- and species-level taxonomic profiles were analyzed to compare responders and non-responders using samples collected after C2D1 (**A**) and at progression (**B**). The heatmap displays the generalized fold change (gFC) values derived from SIAMCAT, and the left annotation provides bacterial categories grouped by functional roles. Asterisks indicate markers with *P* values < 0.05 and a prevalence of ≥10%. *P* values were calculated using the Wilcoxon rank-sum test.

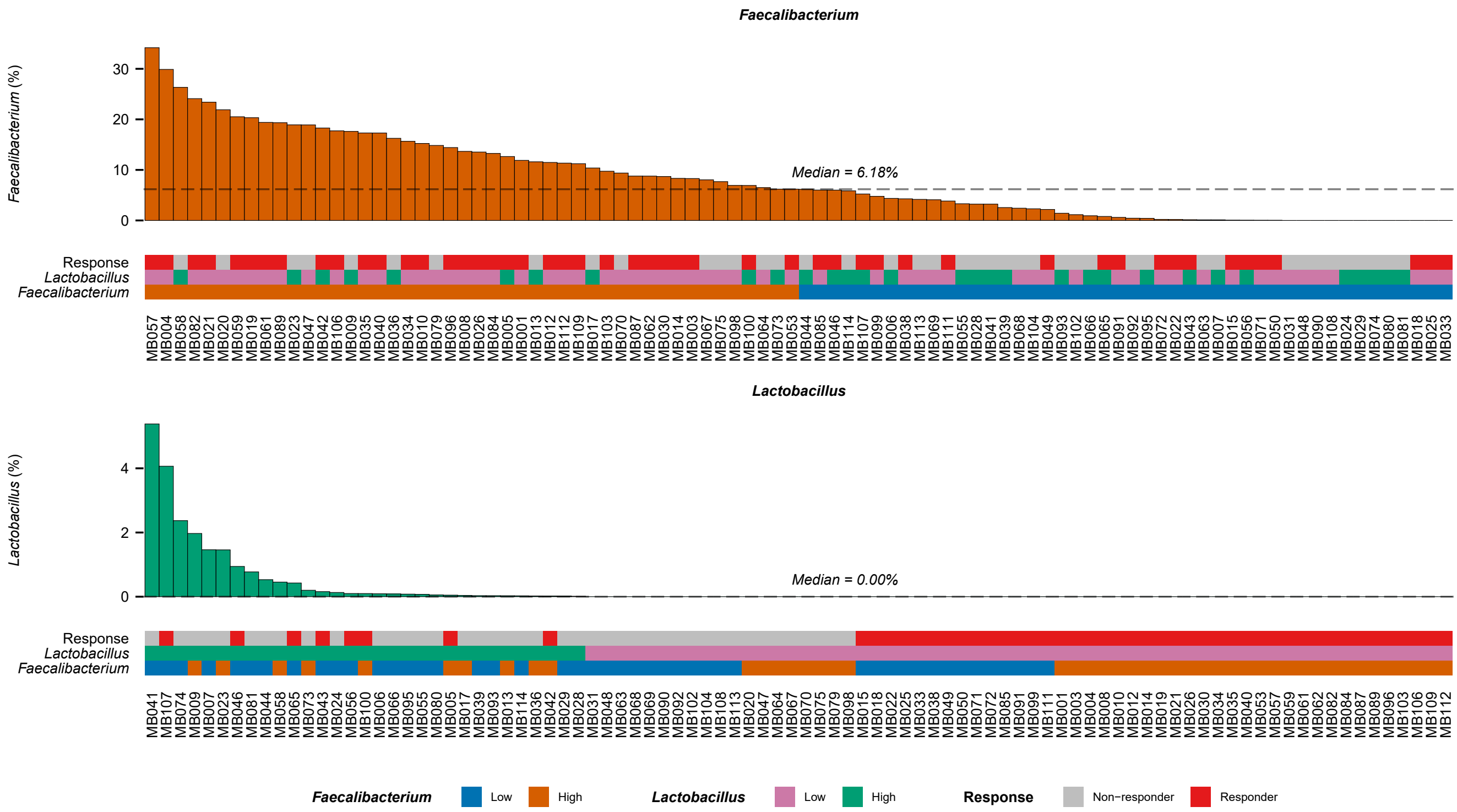

Fig. S6 Distribution of *Faecalibacterium* and *Lactobacillus* abundances. A bar plot shows the abundance of each genus for each patient, sorted by abundance. A heatmap at the bottom of each plot displays patient information. High- and low-abundance groups were defined based on the median abundance of each genus.

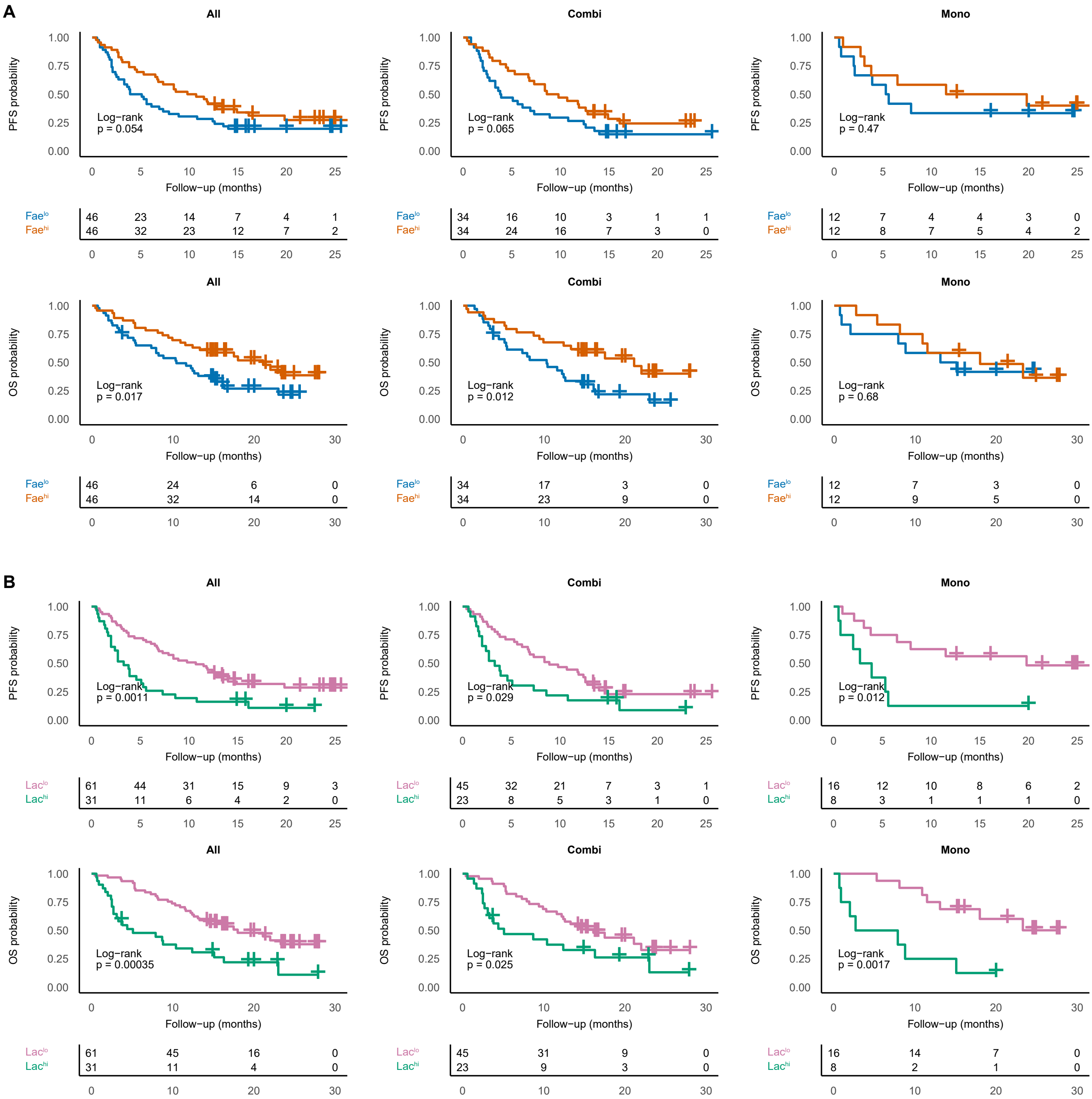

Fig. S7 Survival curves for patients with high and low *Faecalibacterium* abundance (**A**), as well as for patients with high and low *Lactobacillus* abundance (**B**) at screening (cutoff determined by median abundance). *P* values were calculated using the log-rank test.

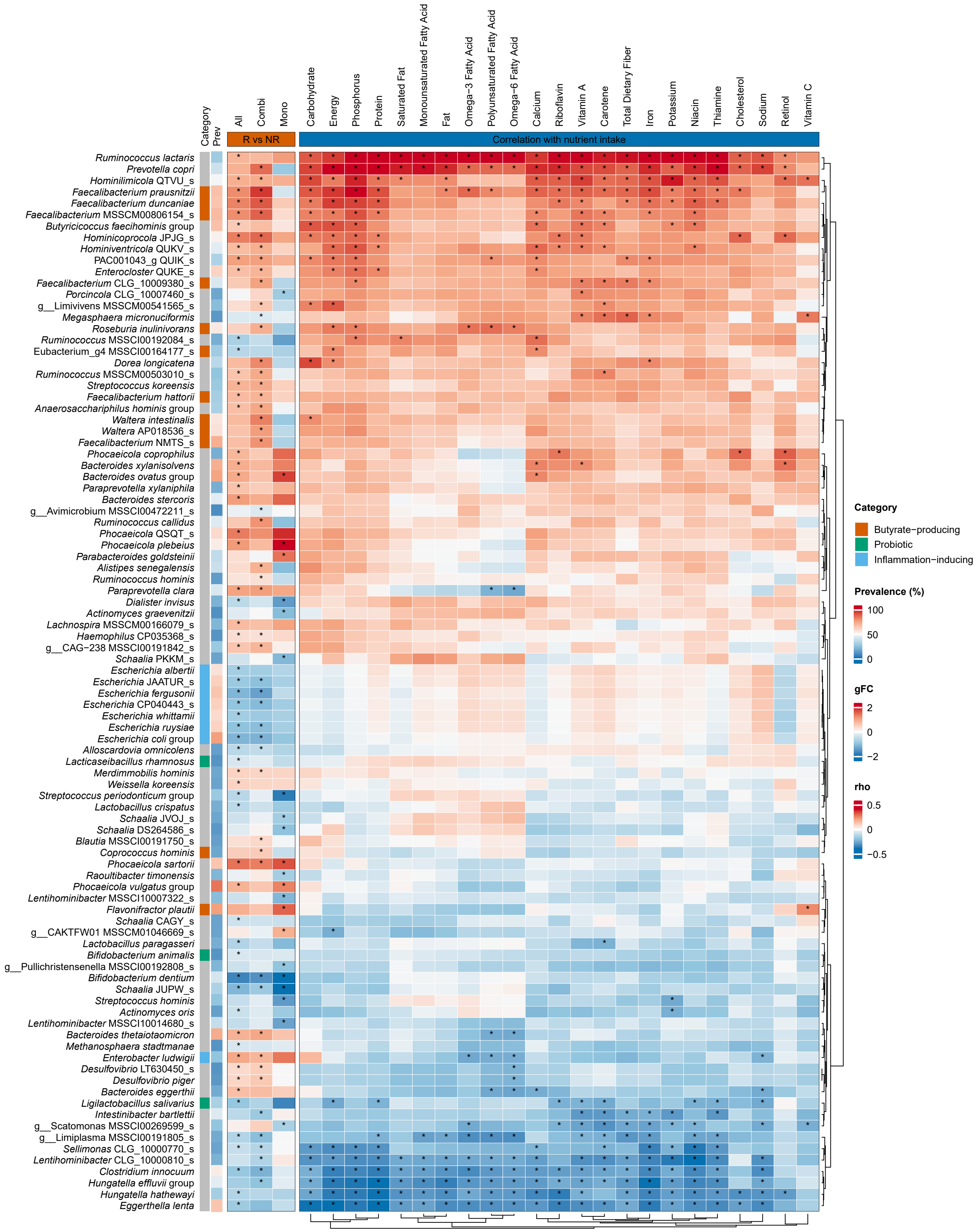

Fig. S8 Taxonomic markers distinguishing responders from non-responders at the species level using screening samples. The heatmap consists of rows corresponding to species-level markers enriched in responders and non-responders. Markers were defined as those with *P* values < 0.05 from the Wilcoxon rank-sum test and a prevalence of ≥10%. The left annotation provides bacterial categories grouped by functional roles and the prevalence of these markers in the cohort. The left heatmap cells show the generalized fold change (gFC) values derived from SIAMCAT, and asterisks indicate the markers. The right heatmap shows Spearman's correlation coefficients (rho) between markers and nutrient intake, and asterisks indicate statistical significance (*P* value < 0.05) as determined by correlation tests.

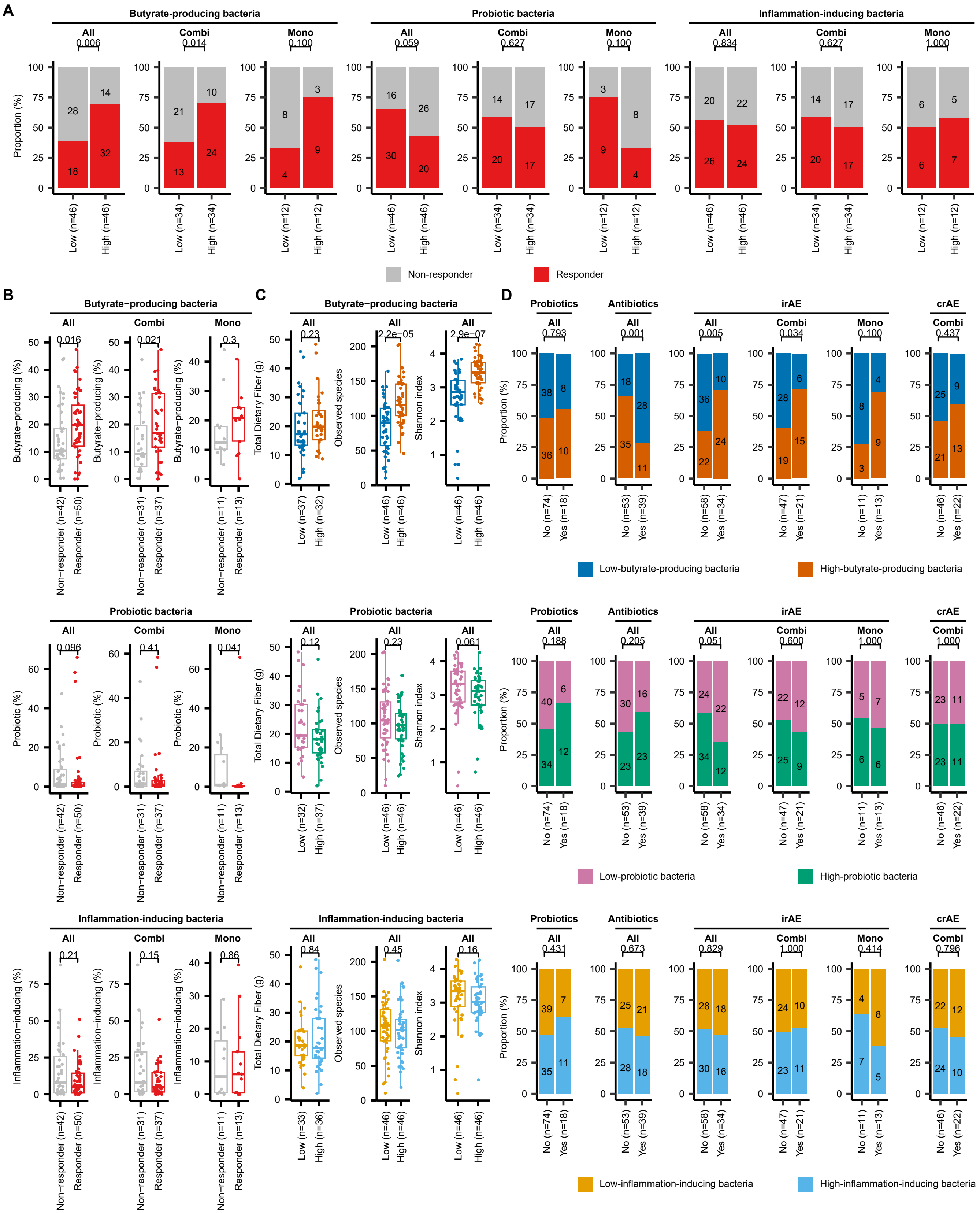

Fig. S9 Association between bacterial categories grouped by functional roles and response. **A** The proportion of responders in the high-abundance group versus the low-abundance group. High- and low-abundance groups were defined based on the median abundance of each bacterial category. **B** Differences in bacterial category abundance between responders and non-responders. **C** Differences in total dietary fiber intake and alpha diversity between the high- and low-abundance groups. **D** Differences in clinical features, including probiotic use, antibiotic use, irAEs, and crAEs, between the high- and low-abundance groups. Shotgun sequencing data were used, except for the alpha diversity analysis, which was performed with amplicon sequencing data. *P* values above the plots were calculated using the Wilcoxon rank-sum test or Fisher's exact test. Results are shown for samples collected at screening.

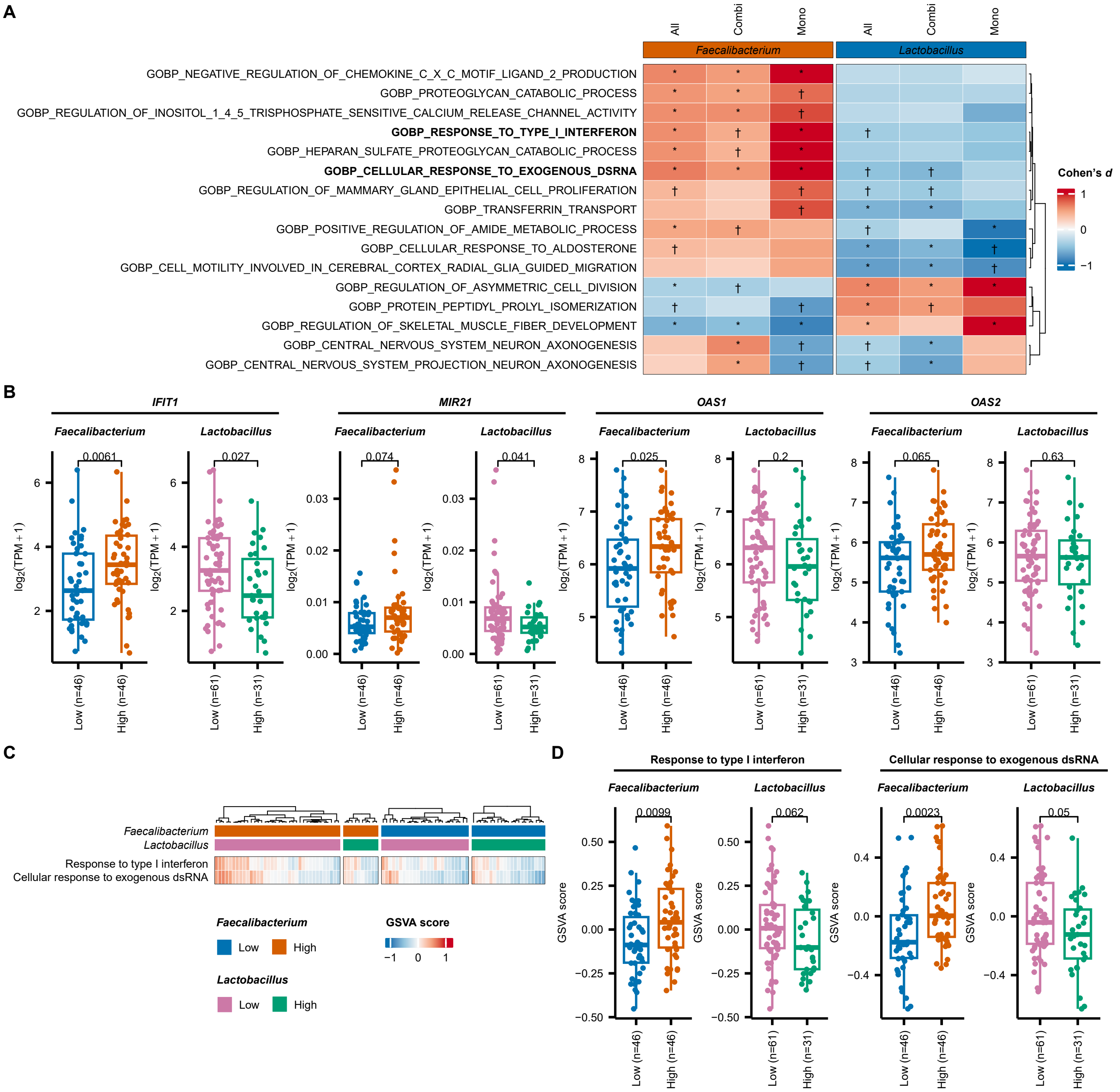

Fig. S10 Transcriptomic signatures associated with *Faecalibacterium* and *Lactobacillus* abundance. **A** The heatmap displays gene sets associated with *Faecalibacterium* and *Lactobacillus* abundance. Each cell shows the Cohen's *d* value for GSVA scores calculated from RNA-seq comparing patients with high versus low genus abundance (stratified at the median). A dagger (†) indicates marginal significance ($P < 0.1$), while an asterisk (*) denotes statistical significance ($P < 0.05$), as calculated using the Wilcoxon rank-sum test. Rows were included if they met all of the following criteria across all comparisons (All, Combi, Mono): (1) marginal significance for either genus; (2) absolute Cohen's *d* > 0.2 for both genera; and (3) opposing directions of effect between *Faecalibacterium* and *Lactobacillus*. **B** Normalized TPM values for type I interferon-related genes were compared between the high- and low-abundance groups. **C** The heatmap displays the GSVA scores for type I interferon-related gene sets across patients, with group annotations shown above. **D** GSVA scores for type I interferon-related gene sets were compared between patients with high and low genus abundance. *P* values above the box plots were calculated using the Wilcoxon rank-sum test.

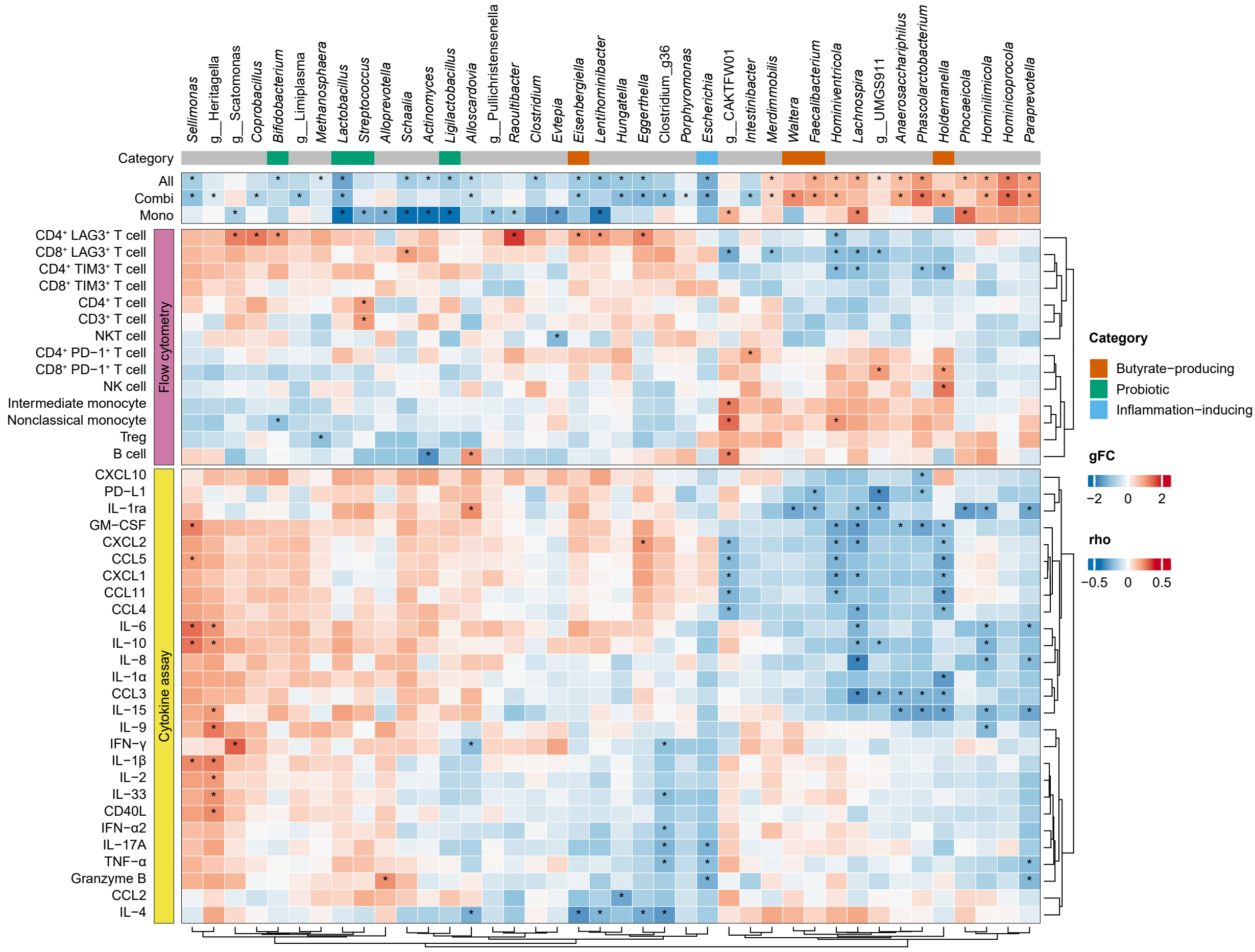

Fig. S11 Associations between genus-level markers and host features at screening. The heatmap consists of columns corresponding to genus-level markers enriched in responders and non-responders at screening. Markers were defined as those with *P* values < 0.05 from the Wilcoxon rank-sum test and a prevalence of ≥10%. The top annotation provides bacterial categories grouped by functional roles. The top heatmap cells show the generalized fold change (gFC) values derived from SIAMCAT, and asterisks indicate the markers. The bottom heatmap shows Spearman's correlation coefficients (rho) between the markers and features from flow cytometry and cytokine assay, with asterisks indicating statistical significance as determined by correlation tests. Rows with at least one statistically significant result were retained.

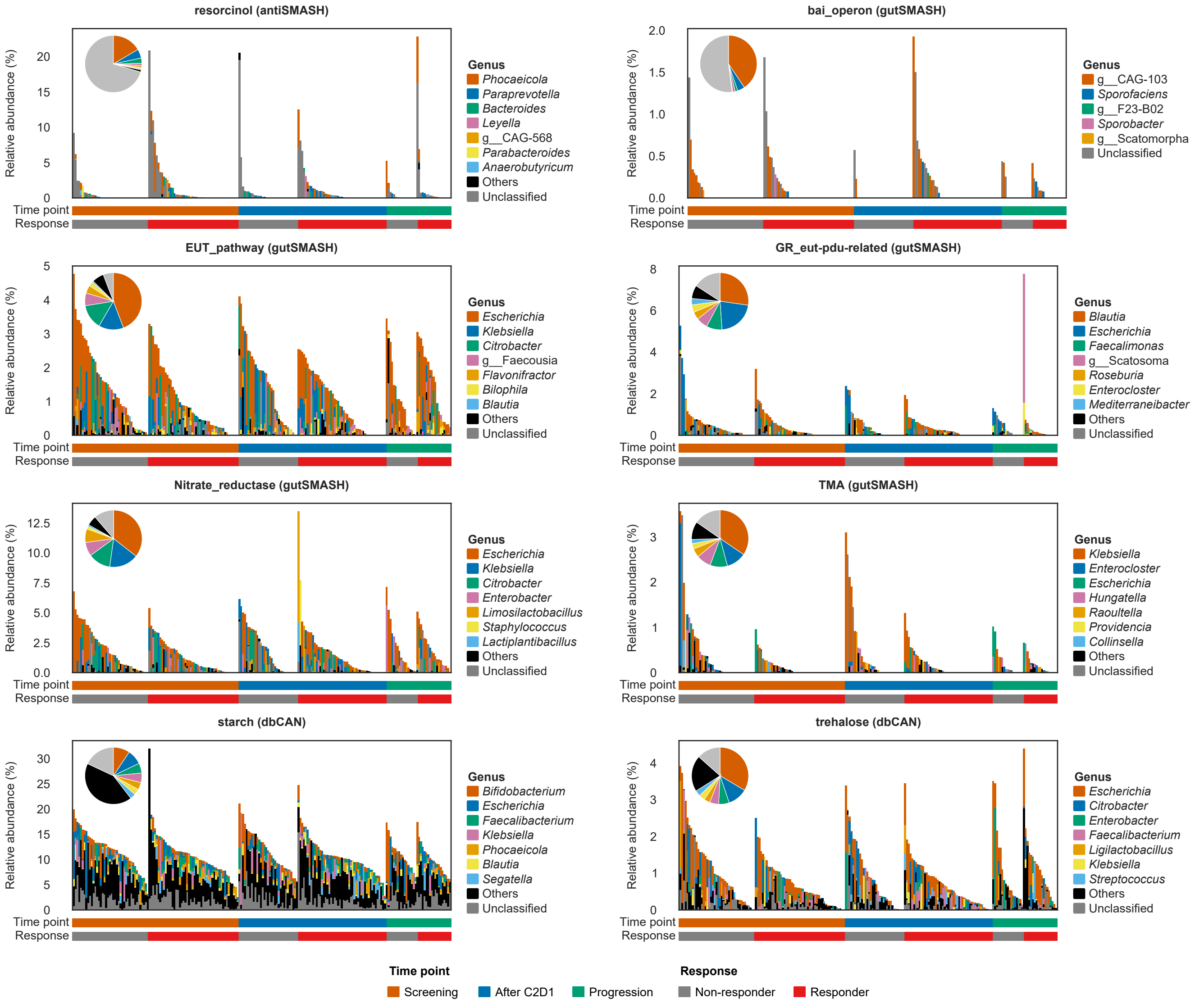

Fig. S12 Microbial gene clusters demonstrating significant differences between responders and non-responders. Bar charts illustrate the relative abundance of the gene clusters, stratified by contributing genera. Each bar represents a single sample, grouped by time point and response status. Within each group, the bars are arranged in order of total abundance. The pie chart in the upper left corner represents the proportion of contributing genera across all samples.

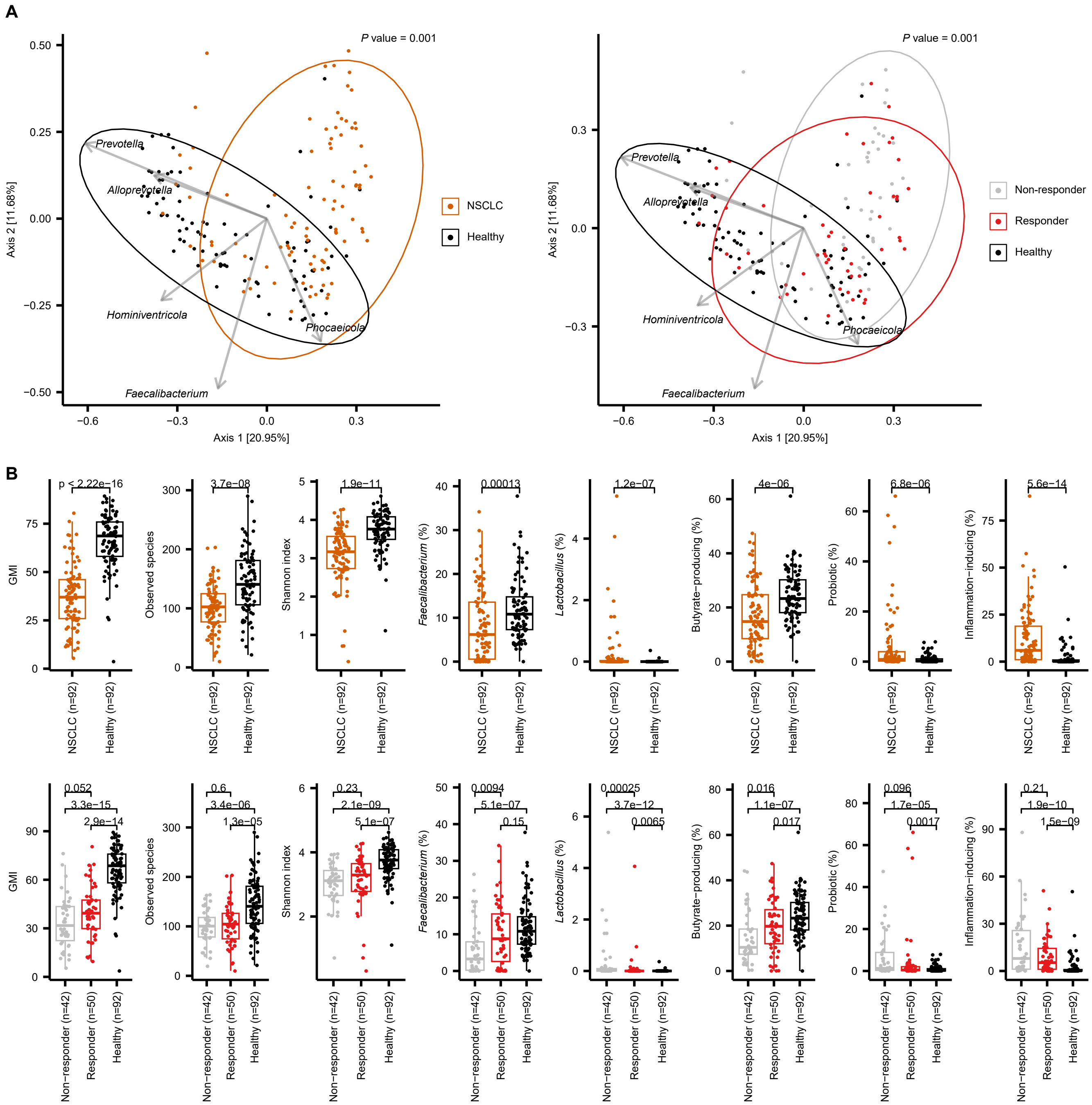

Fig. S13 Comparison of screening samples with those from the healthy Korean cohort. **A** PCoA was performed based on Bray–Curtis dissimilarity at the genus level. Statistical significance was assessed using PERMANOVA to compare groups stratified by cohort or response status. **B** Features including the GMI, alpha diversity, the abundances of *Faecalibacterium* and *Lactobacillus*, and bacterial category abundances were compared between cohorts or response statuses. Shotgun sequencing data were used, except for the GMI calculation and the alpha diversity analysis, for which amplicon sequencing data were used. *P* values above the plots were calculated using the Wilcoxon rank-sum test.

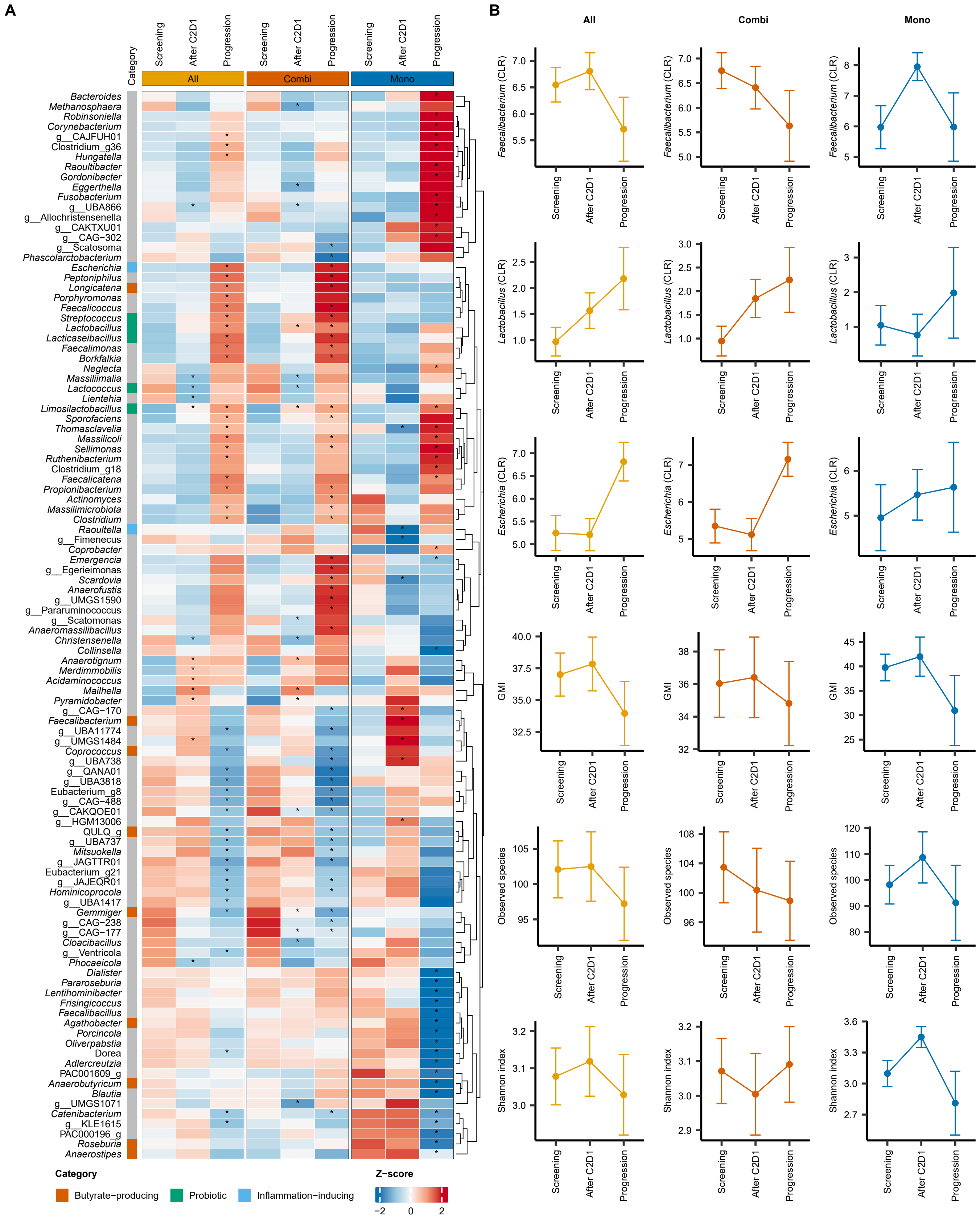

Fig. S14 Temporal changes in microbial features over time. **A** The heatmap displays the Z-score normalized mean values of centered log-ratio (CLR)-transformed abundances of genera, filtered by a prevalence of at least 10% at any one time point. *P* values were obtained from linear mixed-effects models, with time point as a fixed effect and patient as a random effect for each cohort. Asterisks indicate statistical significance (*P* value < 0.05). The left annotation provides bacterial categories grouped by functional roles. Rows with at least one statistically significant result were retained. **B** The mean ± standard error of features, including CLR-transformed abundances of *Faecalibacterium*, *Lactobacillus*, and *Escherichia*, the GMI, and alpha diversity, were plotted for each time point.

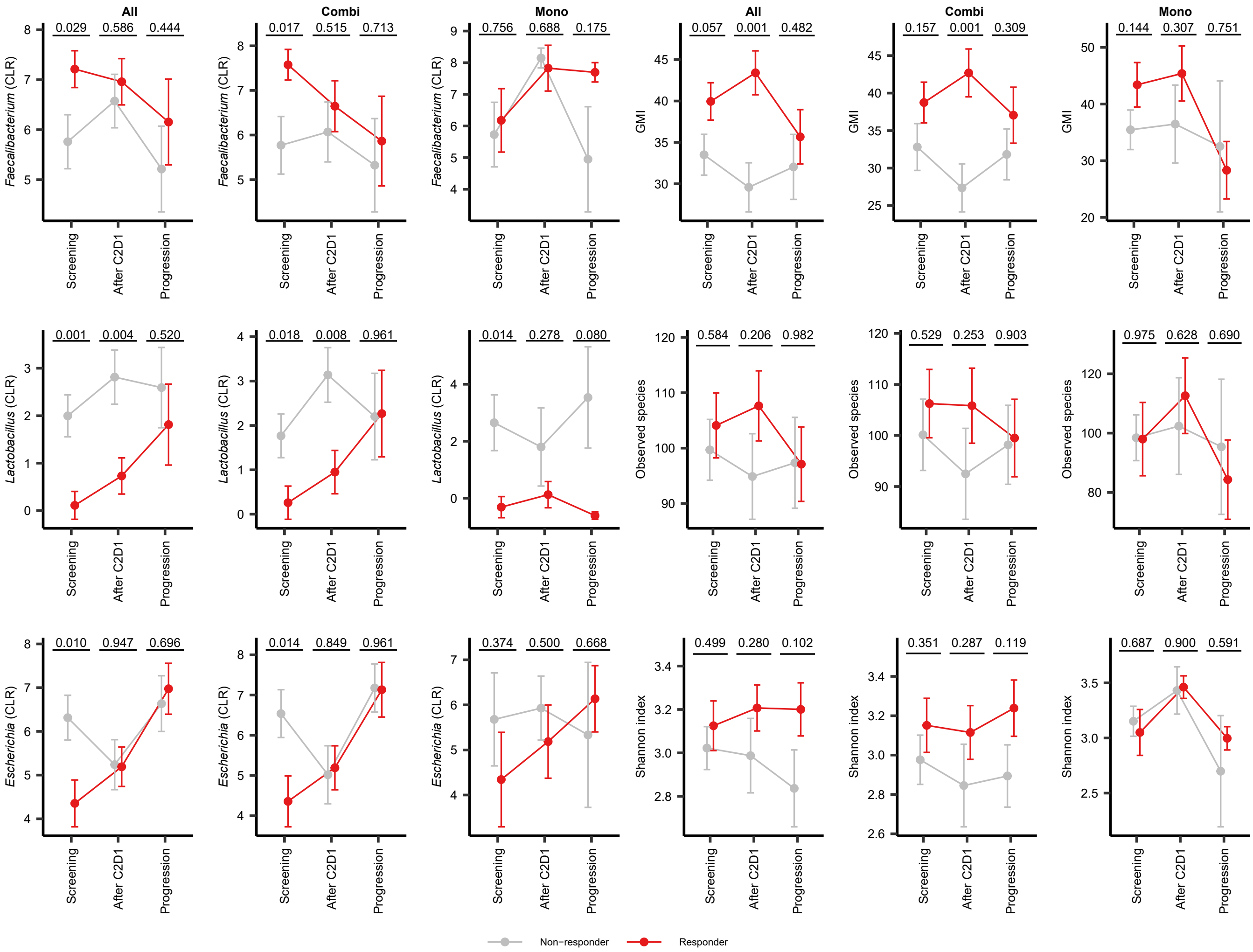

Fig. S15 Temporal changes in microbial features over time, stratified by response status. The mean ± standard error of features, including CLR-transformed abundances of *Faecalibacterium*, *Lactobacillus*, and *Escherichia*, the GMI, and alpha diversity, were plotted for each time point, stratified by response status. *P* values above the plots were calculated using the t-test between responders and non-responders at each time point.

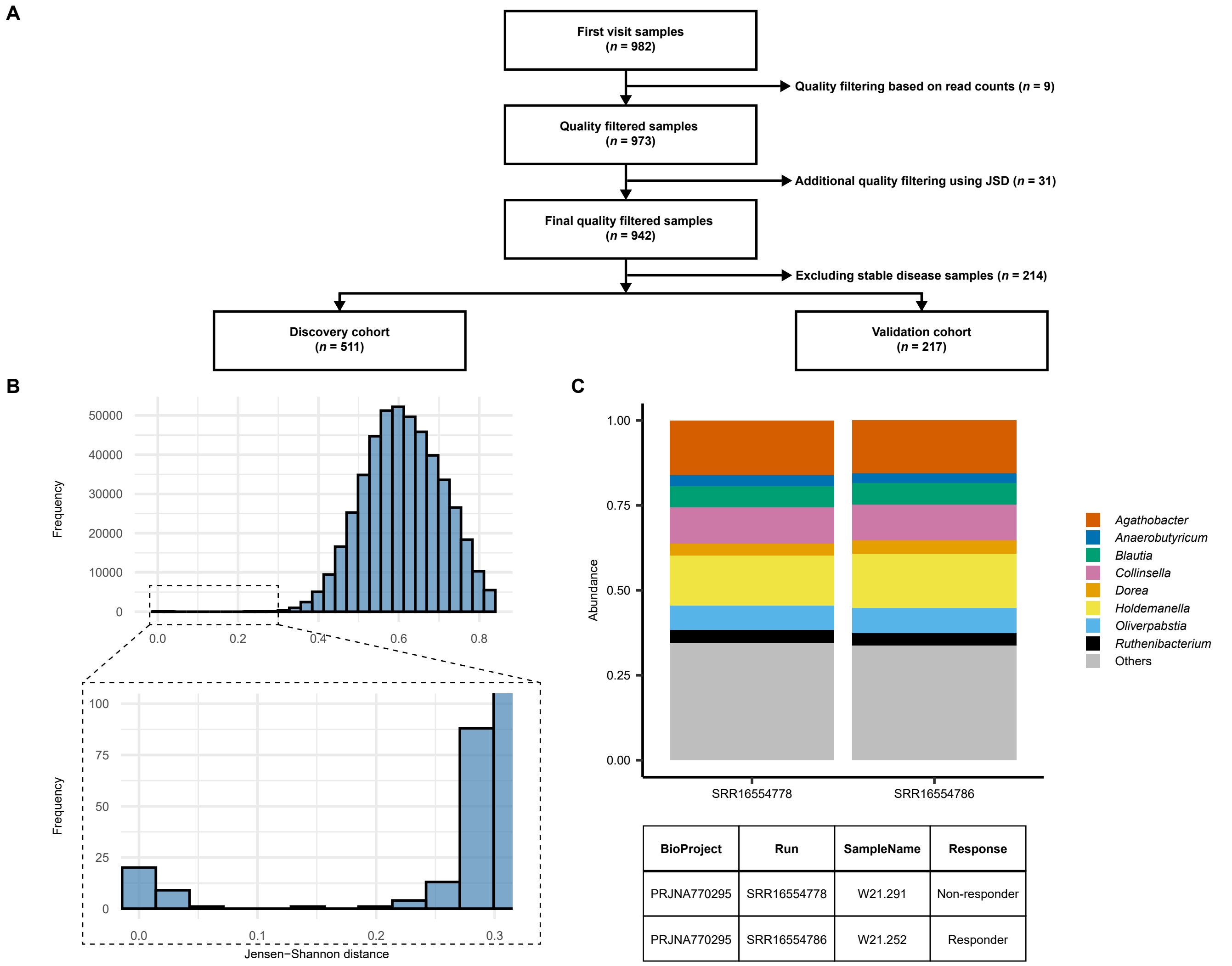

BioProject	Run	SampleName	Response
PRJNA770295	SRR16554778	W21.291	Non-responder
PRJNA770295	SRR16554786	W21.252	Responder

Fig. S16 Quality control process for public metagenomic data. **A** The flow diagram illustrates the quality control process for public metagenomic data. **B** A histogram shows the Jensen–Shannon distance (JSD) at the genus level between pairs of quality-filtered samples based on read counts (cutoff: 150,000). A threshold of 0.1 for JSD was used for filtering. **C** The taxonomic composition at the genus level is shown for SRR16554778 and SRR16554786 (see Methods).

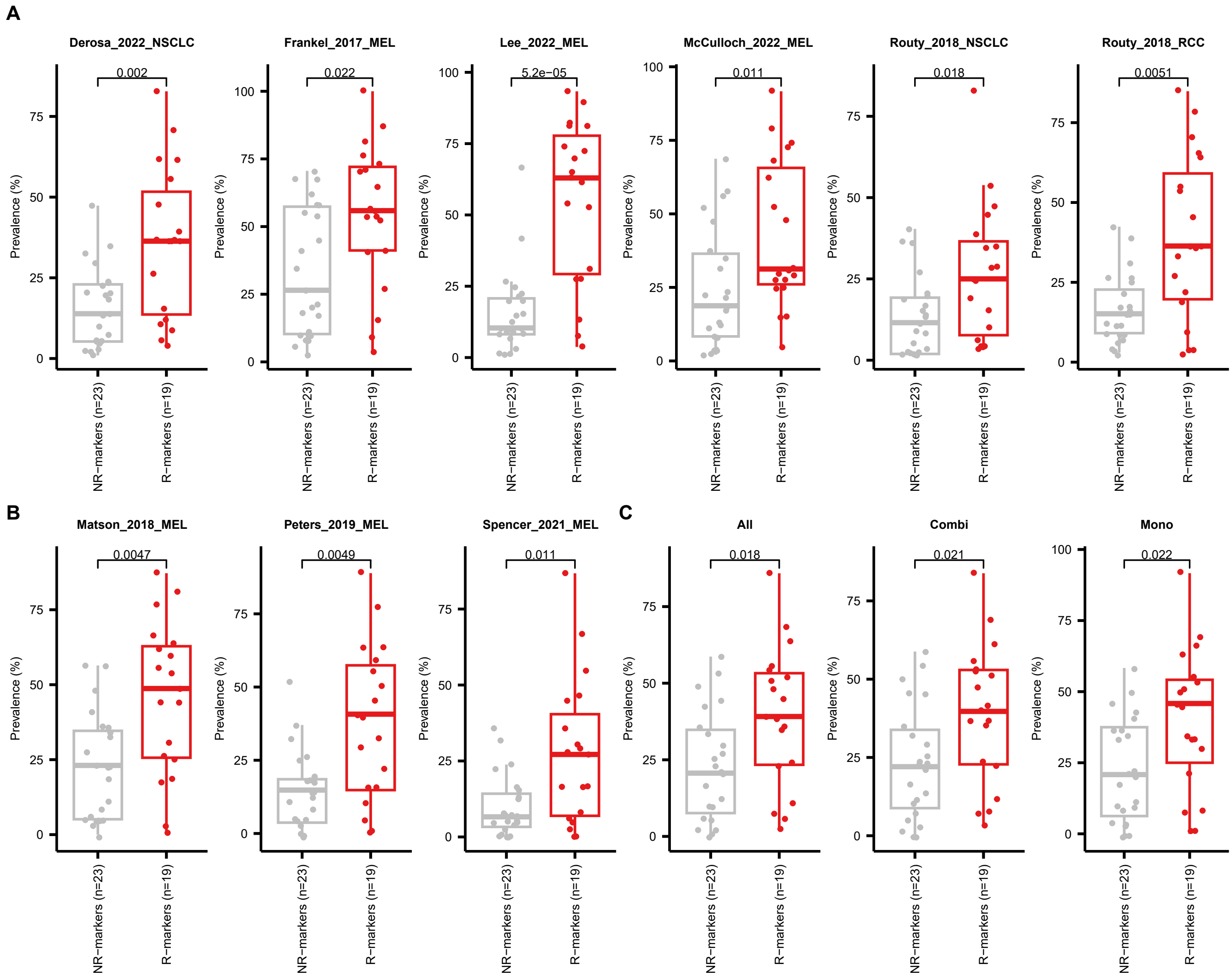

Fig. S17 Prevalence of species-level meta-analysis markers distinguishing responders from non-responders in the discovery cohorts. Prevalence was calculated in the discovery cohorts (**A**), the validation cohorts (**B**), and the Korean NSCLC cohort (**C**). The *P* values shown above the plots were calculated using the Wilcoxon rank-sum test. R indicates responders, whereas NR indicates non-responders.

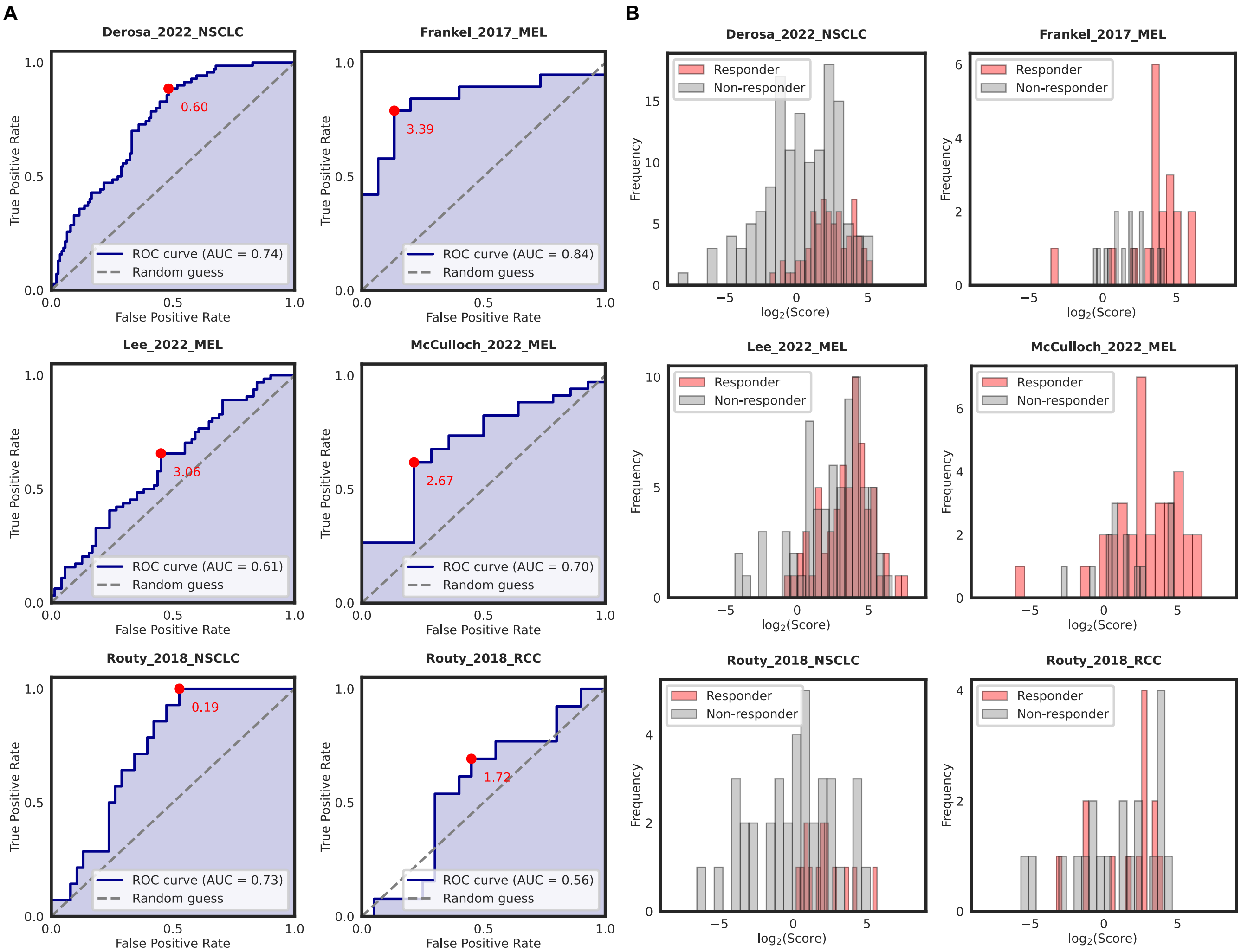

Fig. S18 Assessment of response using the bacterial ratio score in the discovery cohorts. **A** ROC curves and AUC values derived from the bacterial ratio score in the discovery cohorts. The red point on the ROC curve indicates the threshold that maximizes Youden's index, and the adjacent red label shows the threshold after the $\log_2$ transformation. **B** Distributions of the bacterial ratio score in the discovery cohorts.

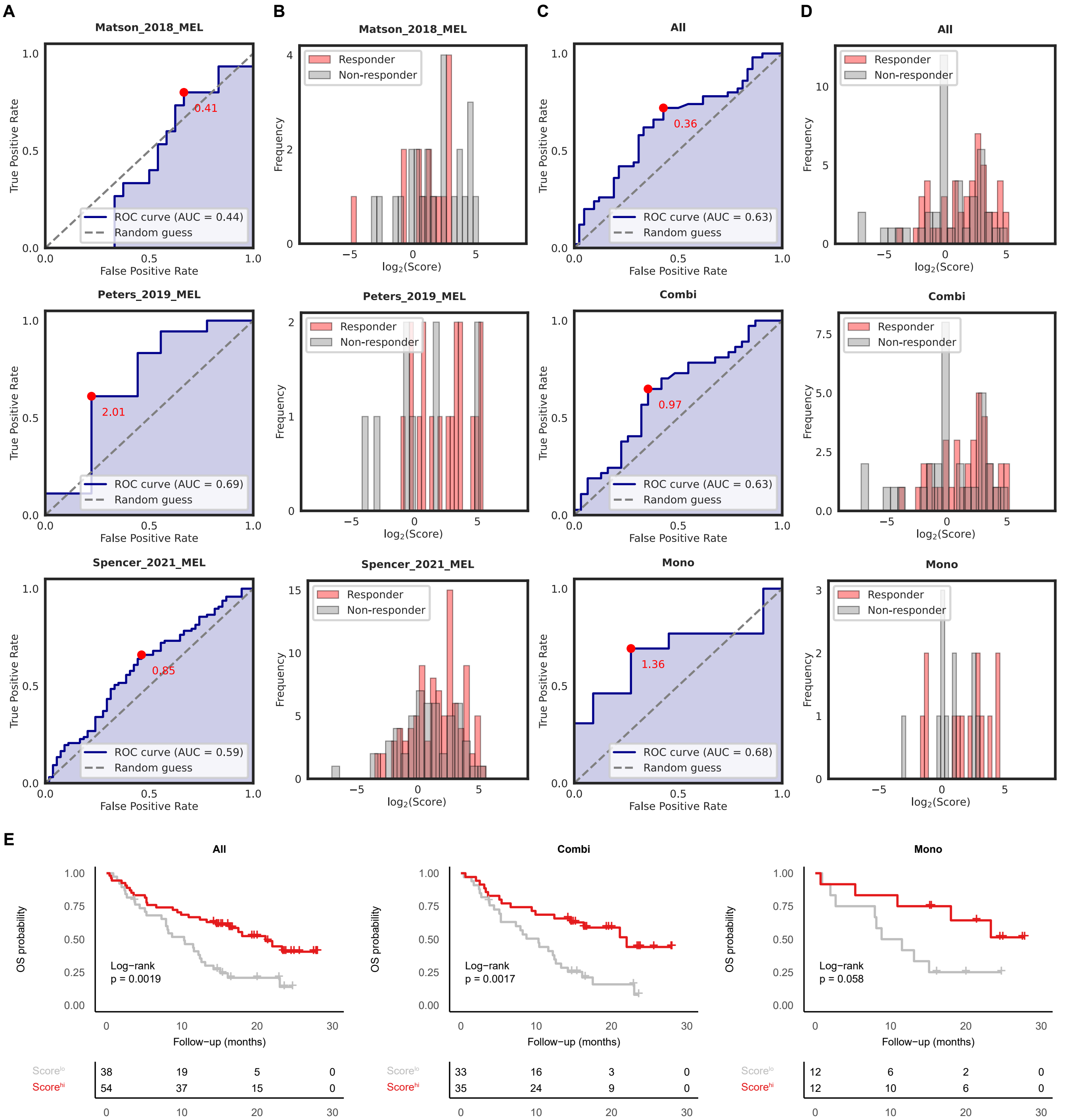

Fig. S19 Assessment of response using the bacterial ratio score in the validation cohorts and the Korean NSCLC cohort. **A** ROC curves and AUC values derived from the bacterial ratio score in the validation cohorts. **B** Distributions of the bacterial ratio score in the validation cohorts. **C** ROC curves and AUC values derived from the bacterial ratio score in the Korean NSCLC cohort. **D** Distributions of the bacterial ratio score in the Korean NSCLC cohort. **E** Survival curves for patients with high and low bacterial ratio scores in the Korean NSCLC cohort. The red point on the ROC curve indicates the threshold that maximizes Youden's index, and the adjacent red label shows the threshold after the $\log_2$ transformation. High and low groups were defined using the optimal threshold based on Youden's index, and only first-visit samples were used. *P* values were calculated using the log-rank test.

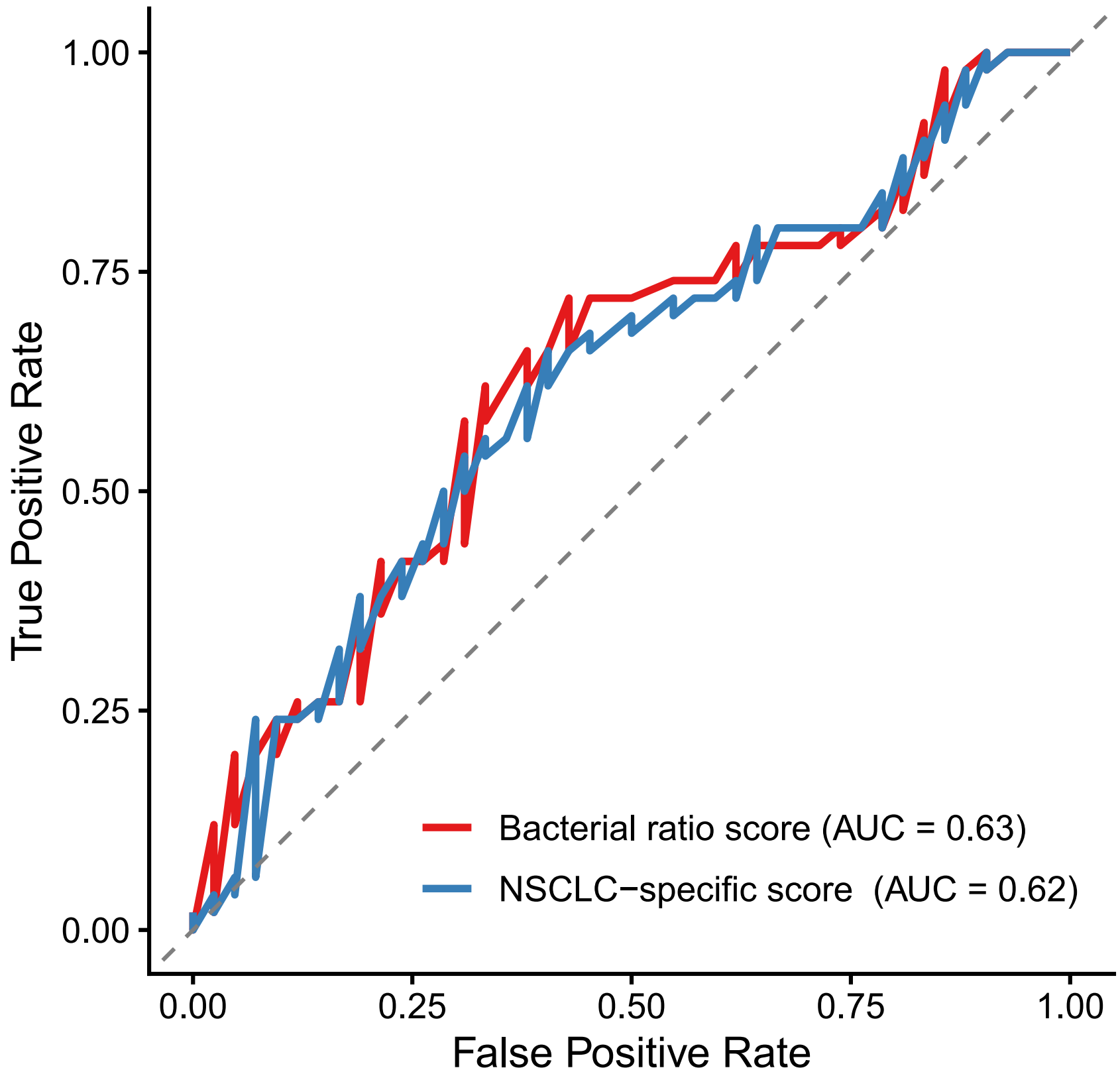

Fig. S20 Comparison of the bacterial ratio score and the NSCLC-specific score in the Korean NSCLC cohort. ROC curves and AUC values for the bacterial ratio score (derived from six discovery cohorts) and the NSCLC-specific score (derived from two NSCLC public cohorts, Derosa_2022_NSCLC and Routy_2018_NSCLC) applied to the Korean NSCLC cohort (*n* = 92).

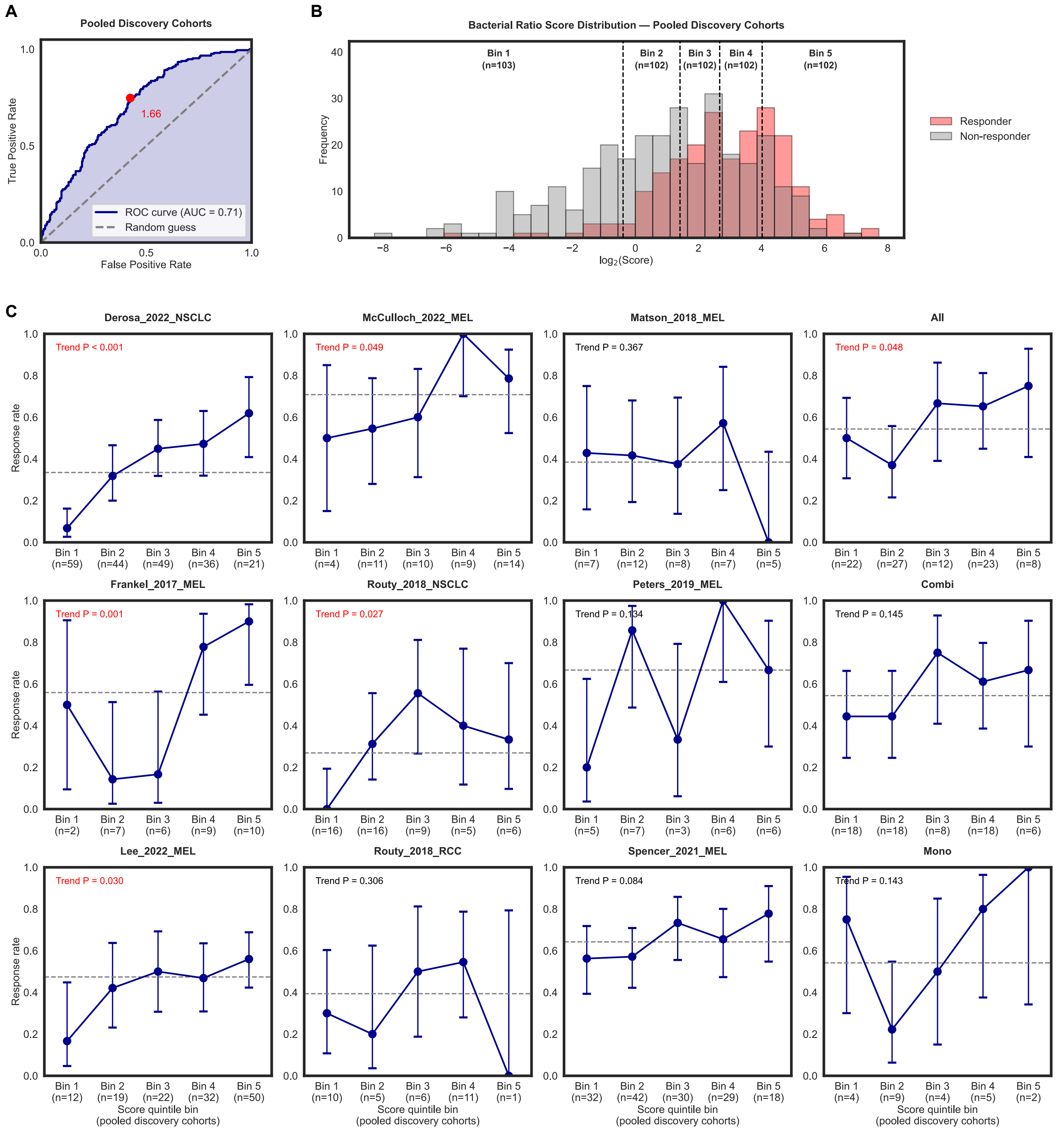

Fig. S21 Derivation and application of a fixed threshold and quintile bins for the bacterial ratio score using pooled discovery cohorts. **A** ROC curve and AUC value derived from the bacterial ratio score in the pooled discovery cohorts (n = 511). The red point on the ROC curve indicates the threshold that maximizes Youden's index, and the adjacent red label shows the threshold after the $\log_2$ transformation. **B** Distribution of the bacterial ratio score in the pooled discovery cohorts, stratified by response status. Dashed vertical lines indicate the boundaries of the five score quintile bins; bin labels show the number of patients per bin. **C** Response rate by score quintile bin across the discovery, validation, and Korean NSCLC cohorts. Quintile bin boundaries were fixed using the pooled discovery cohorts and applied unchanged to all cohorts. Error bars represent 95% Wilson confidence intervals; dashed horizontal lines indicate the response rate for each cohort. Cochran–Armitage trend test P values are shown in red when P < 0.05.

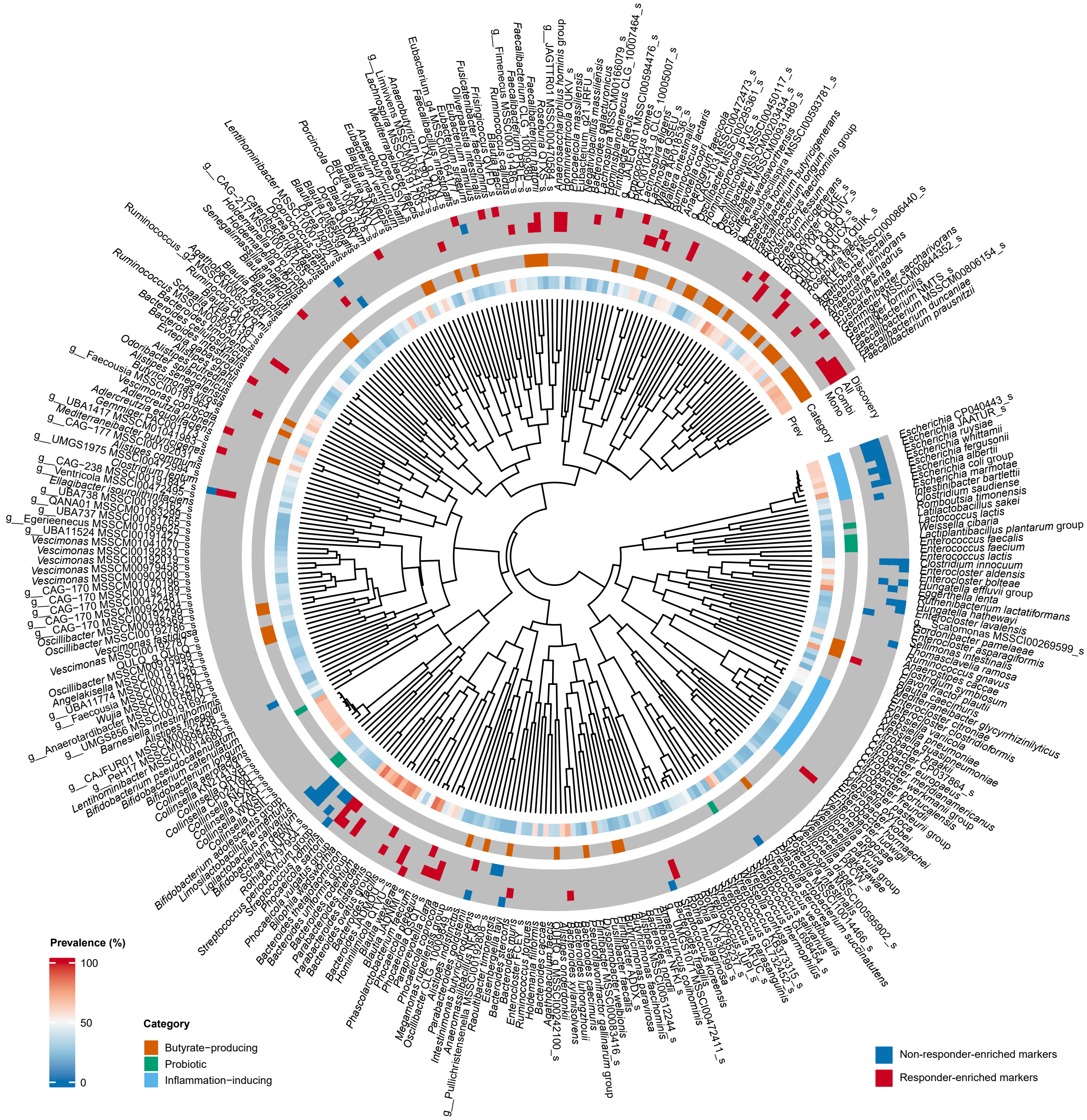

Fig. S22 Relationship between the public markers and the Korean markers. Species were filtered from data sampled at screening from all patients (n = 92) based on a prevalence threshold of at least 20%, yielding 306 species. Spearman's correlation coefficients were calculated, and distances were defined as (1 - Spearman's correlation coefficient). Hierarchical clustering using the complete linkage method was applied to group species, and a dendrogram was generated. The outer heatmap shows the significant markers identified in the discovery cohorts (P value < 0.05 based on a random-effects meta-analysis, present in all six cohorts) and in the Korean NSCLC cohort (P value < 0.05 based on the Wilcoxon rank-sum test, with a prevalence ≥ 10%). The middle heatmap displays bacterial categories grouped by functional roles, and the inner heatmap depicts prevalence in samples collected at screening from all patients in the Korean NSCLC cohort.

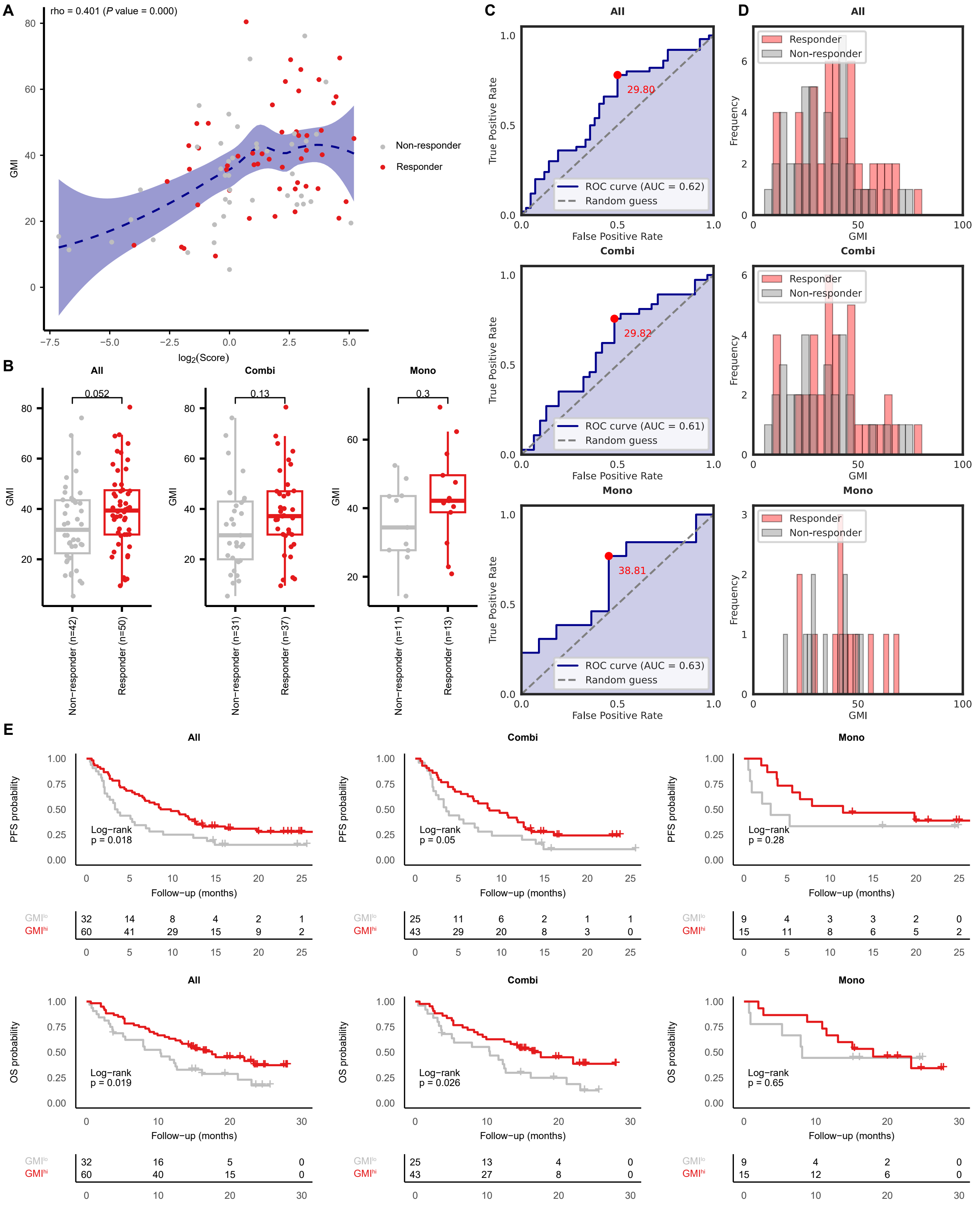

Fig. S23 Assessment of response using the GMI. **A** A scatter plot illustrating the relationship between the bacterial ratio score and the GMI. Each point is color-coded by response status, and a dashed line represents a smoothing curve with a 95% confidence interval. Spearman's correlation coefficient (rho) is displayed in the top-left corner. **B** Differences in the GMI between responders and non-responders. The *P* values shown above the plots were calculated using the Wilcoxon rank-sum test. **C** ROC curves and AUC values based on the GMI in the Korean NSCLC cohort. The red point on the ROC curve indicates the threshold that maximizes Youden's index, and the adjacent red label shows the threshold. **D** Distribution patterns of the GMI in the Korean NSCLC cohort. **E** Survival curves comparing patients with high and low GMI in the Korean NSCLC cohort. High and low groups were defined using the optimal threshold based on Youden's index, with only first-visit samples included. *P* values were calculated via the log-rank test.

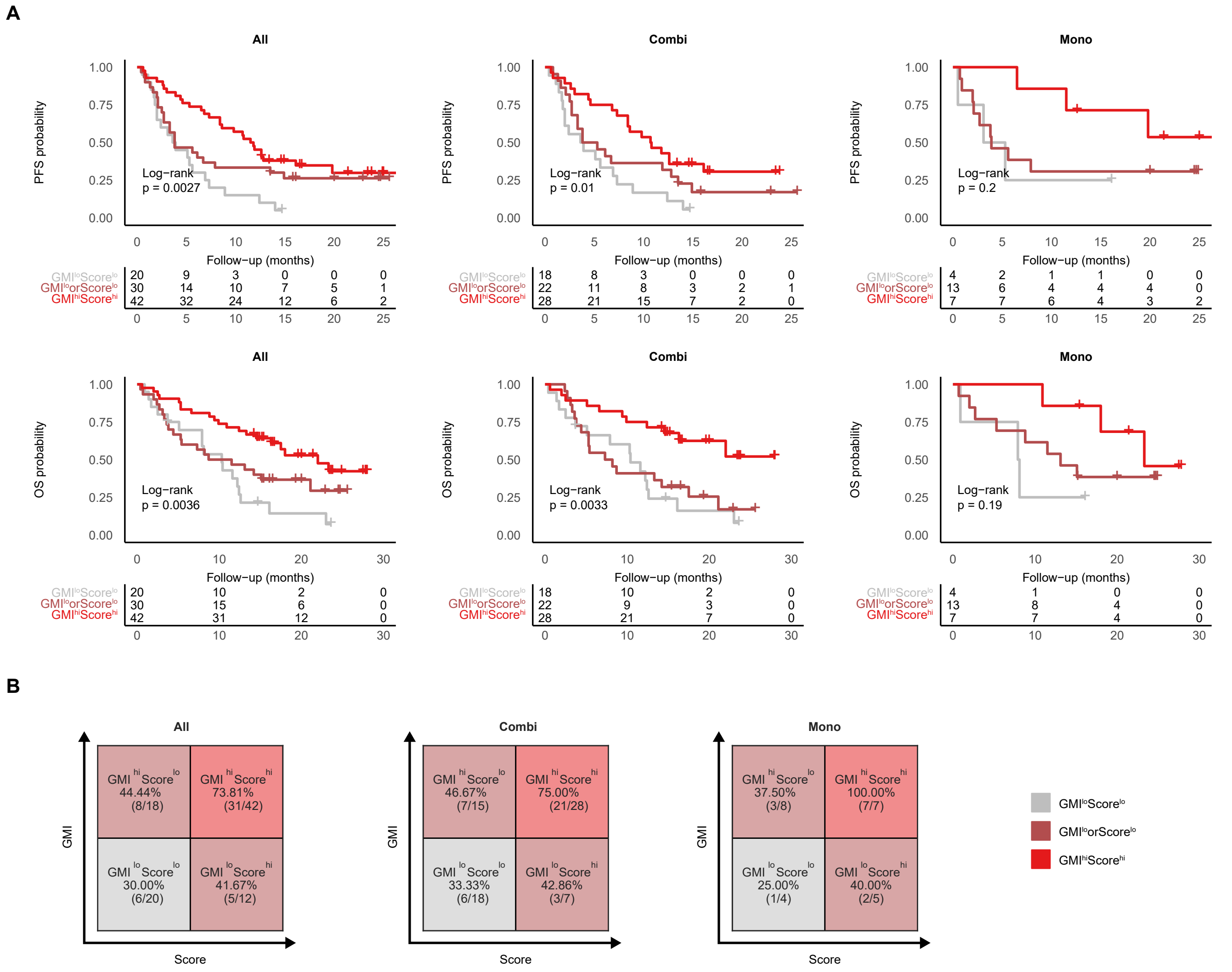

Fig. S24 Utilization of GMI and bacterial ratio score to stratify response to ICI in the Korean NSCLC cohort. **A** Survival curves comparing patients grouped by combined GMI and the bacterial ratio score: both high, both low, or discordant (high/low or low/high) in the Korean NSCLC cohort. High and low groups were defined using the optimal threshold based on Youden's index, with only first-visit samples included. *P* values were calculated via the log-rank test. **B** Proportion of responders in each group stratified by combined GMI and the bacterial ratio score.

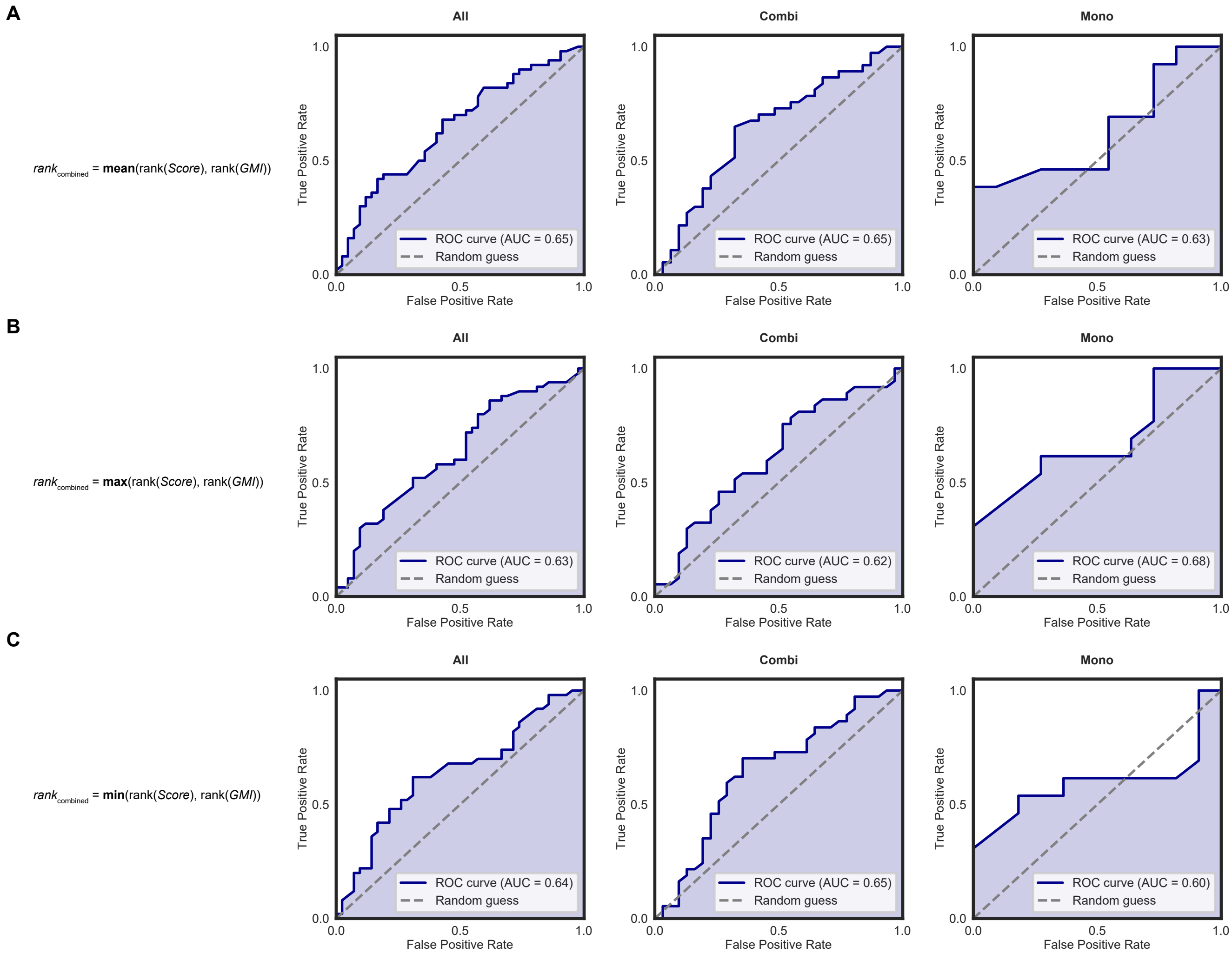

Fig. S25 Assessment of response using rank-based fusion of GMI and the bacterial ratio score in the Korean NSCLC cohort. For each patient, GMI and the bacterial ratio score were independently converted to ranks, with higher ranks corresponding to higher values. A combined rank was then derived using one of three fusion strategies: the mean of the two ranks (**A**); the maximum of the two ranks (**B**), representing a permissive approach where a high value in either metric elevates the combined rank; or the minimum of the two ranks (**C**), reflecting a conservative approach that requires high values in both metrics. ROC curves were generated for each of the three rank-based fusion methods.

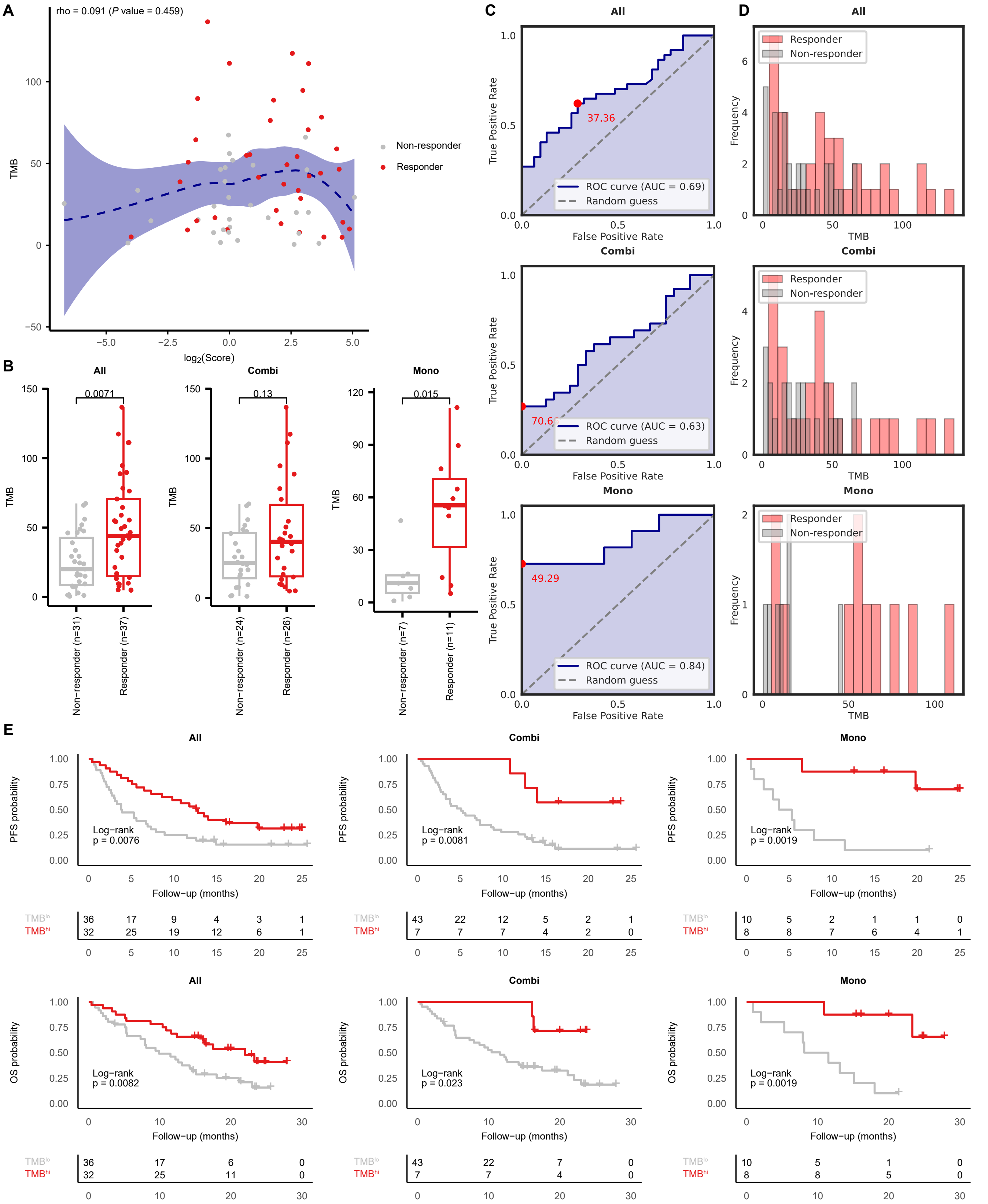

Fig. S26 Assessment of response using TMB. **A** A scatter plot illustrating the relationship between the bacterial ratio score and TMB. Each point is color-coded by response status, and a dashed line represents a smoothing curve with a 95% confidence interval. Spearman's correlation coefficient (rho) is displayed in the top-left corner. **B** Differences in TMB between responders and non-responders. The *P* values shown above the plots were calculated using the Wilcoxon rank-sum test. **C** ROC curves and AUC values based on TMB in the Korean NSCLC cohort. The red point on the ROC curve indicates the threshold that maximizes Youden's index, and the adjacent red label shows the threshold. **D** Distribution patterns of TMB in the Korean NSCLC cohort. **E** Survival curves comparing patients with high and low TMB in the Korean NSCLC cohort. High and low groups were defined using the optimal threshold based on Youden's index, with only first-visit samples included. *P* values were calculated via the log-rank test.

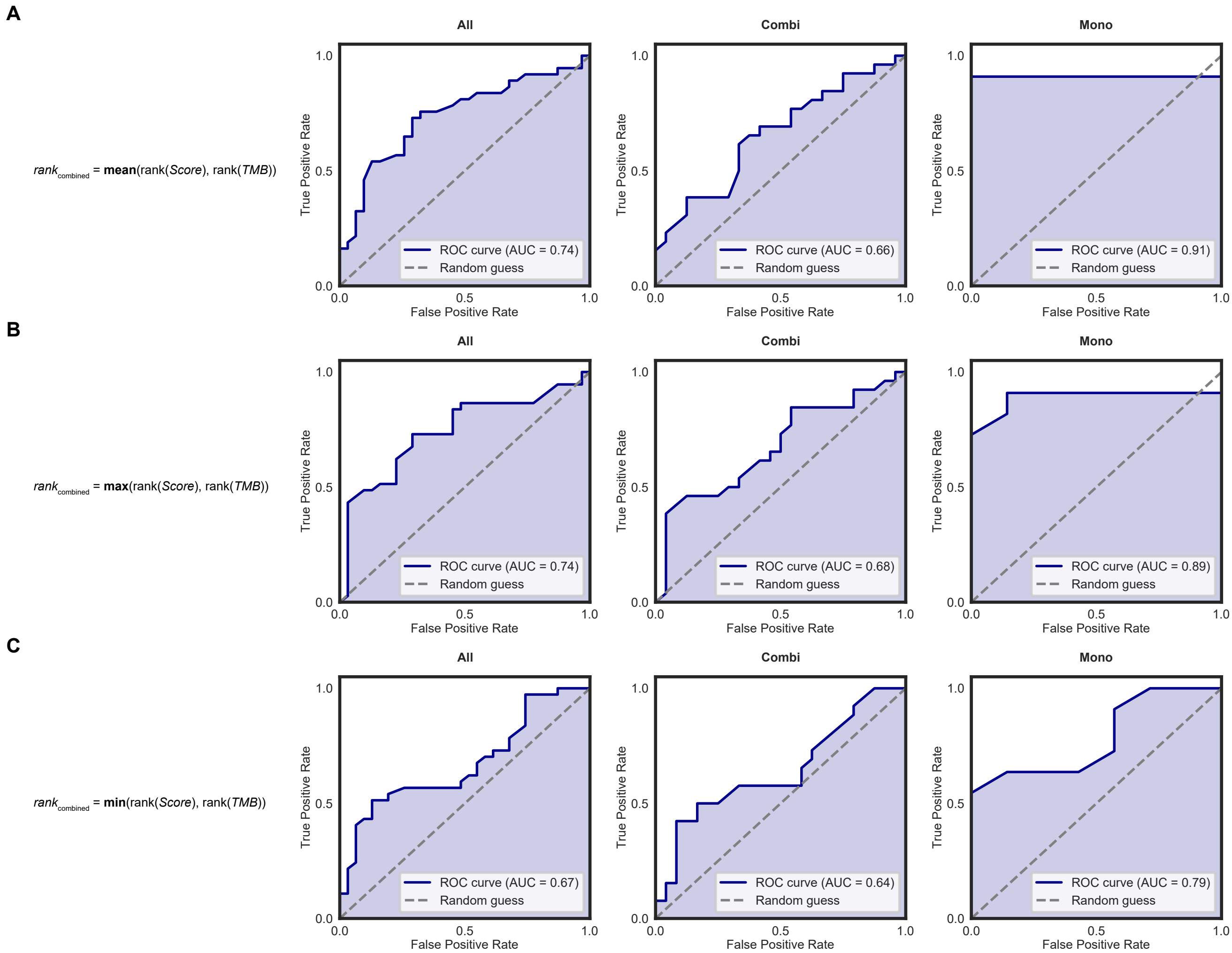

Fig. S27 Assessment of response using rank-based fusion of TMB and the bacterial ratio score in the Korean NSCLC cohort. For each patient, TMB and the bacterial ratio score were independently converted to ranks, with higher ranks corresponding to higher values. A combined rank was then derived using one of three fusion strategies: the mean of the two ranks (**A**); the maximum of the two ranks (**B**), representing a permissive approach where a high value in either metric elevates the combined rank; or the minimum of the two ranks (**C**), reflecting a conservative approach that requires high values in both metrics. ROC curves were generated for each of the three rank-based fusion methods.
